# Mutation of S461, in the GOLGA3 phosphorylation site, does not affect mouse spermatogenesis

**DOI:** 10.7717/peerj.15133

**Published:** 2023-04-17

**Authors:** Changtong Xu, Mingcong Zhu, Shuqin Zhao, Xin Zhang, Ying Wang, Mingxi Liu

**Affiliations:** 1 State Key Laboratory of Reproductive Medicine, Department of Histology and Embryology, School of Basic Medical Sciences, Nanjing Medical University, Nanjing, China; 2 State Key Laboratory of Reproductive Medicine, Department of Reproduction, Women’s Hospital of Nanjing Medical University, Nanjing Maternity and Child Health Care Hospital, Nanjing, China; 3 State Key Laboratory of Reproductive Medicine, The Affiliated Taizhou People’s Hospital of Nanjing Medical University, Taizhou School of Clinical Medicine, Nanjing Medical University, Nanjing, China

**Keywords:** Golgin subfamily A member 3 (Golga3), Protein phosphorylation, Spermatogenesis, Male fertility

## Abstract

**Background:**

Golgin subfamily A member 3 (*Golga3*), a member of the golgin subfamily A, is highly expressed in mouse testis. The GOLGA3 protein, which contains eight phosphorylation sites, is involved in protein transport, cell apoptosis, Golgi localization, and spermatogenesis. Although it has been previously reported that nonsense mutations in *Golga3* cause multiple defects in spermatogenesis, the role of *Golga3* in the testis is yet to be clarified.

**Methods:**

Immunofluorescence co-localization in cells and protein dephosphorylation experiments were performed.* Golga3*^ S461L/S461L^mice were generated using cytosine base editors. Fertility tests as well as computer-assisted sperm analysis (CASA) were then performed to investigate sperm motility within caudal epididymis. Histological and immunofluorescence staining were used to analyze testis and epididymis phenotypes and TUNEL assays were used to measure germ cell apoptosis in spermatogenic tubules.

**Results:**

Immunofluorescence co-localization showed reduced Golgi localization of GOLGA3^S465L^ with some protein scattered in the cytoplasm of HeLa cells .In addition, protein dephosphorylation experiments indicated a reduced band shift of the dephosphorylated GOLGA3^S465L^, confirming S461 as the phosphorylation site. *Golga3* is an evolutionarily conserved gene and* Golga3*^S461L/S461L^mice were successfully generated using cytosine base editors. These mice had normal fertility and spermatozoa, and did not differ significantly from wild-type mice in terms of spermatogenesis and apoptotic cells in tubules.

**Conclusions:**

*Golga3* was found to be highly conserved in the testis, and GOLGA3 was shown to be involved in spermatogenesis, especially in apoptosis and Golgi complex-mediated effects. Infertility was also observed in *Golga3* KO male mice. Although GOLGA3^S465L^showed reduced localization in the Golgi with some expression in the cytoplasm, this abnormal localization did not adversely affect fertility or spermatogenesis in male C57BL/6 mice. Therefore, mutation of the S461 GOLGA3 phosphorylation site did not affect mouse spermatogenesis.

## Introduction

Approximately 15% of couples around the world suffer from infertility, with male infertility factors accounting for 30%–50% of all cases (Sharlip et al., 2002; Thirumavalavan, Gabrielsen & Lamb, 2019; Winters & Walsh, 2014). Male infertility is mainly caused by disorders of spermatogenesis that are clinically manifested as azoospermia, teratospermia or different degrees of asthenospermia and oligozoospermia (Yang, Li & Li, 2021). These may result from genetic factors, estimated to account for at least 15% of male infertility (Krausz & Riera-Escamilla, 2018). The widespread use of CRISPR-Cas9 gene editing and base editors in eukaryotic cells has led to rapid advances in genomics and proteomics research. These gene-editing techniques have been used to generate mouse models to screen and identify specific genes that influence male reproductive development (Gao et al., 2021; Gaudelli et al., 2017; Li et al., 2013; Shen et al., 2013). Genes associated with spermatogenesis are of great significance for exploring the mechanisms of mitosis, meiosis and spermatogenesis. At the same time, by providing a reference for subsequent research, genes that are not essential for spermatogenesis prevent researchers from wasting time and resources on factors that do not contribute to significant reproductive phenotypes (Castaneda et al., 2017; Liu et al., 2021; Wu et al., 2021; Wu et al., 2022; Zhang et al., 2021a; Zhang et al., 2021b).

Four main stages of spermatogenesis are recognized: (I) primary spermatocytes undergo mitotic proliferation and spermatogonia proliferate and differentiate differentiation; (II) Secondary spermatocytes produce round sperm cells during meiosis; (III) Round sperm cells transform into elongated sperm cells; (IV) After maturation, the sperm are stored in the lumen of the spermatogenic tubules (Babakhanzadeh et al., 2020; De Kretser et al., 1998). Spermatogenesis is a complex process that is modulated through various protein modifications, including methylation, acetylation, phosphorylation, ubiquitination, palmitoylation, etc (Berruti, 2021; De Kretser et al., 1998; Inoue et al., 2014; Shiota et al., 2018; Wu et al., 2010; Wu et al., 2022). Among these, phosphorylation plays a significant role in spermatogenesis by regulating protein structure and activity, with previous analyses identifying 8,187 different phosphopeptides, corresponding to 2,661 individual phosphoproteins, during full spermatogenesis in the human testis. In human testicular tissues, phosphorylation mainly regulates chromosome organization, DNA packaging, cell cycle, RNA splicing, and cellular stress response (Castillo et al., 2019; Chen et al., 2021; Inoue et al., 2014; Kavarthapu et al., 2019; Walczak & Heald, 2008).

As a member of Golgin subfamily A, GOLGA3 represents a Golgi complex-associated protein that is involved in protein transport, apoptosis, Golgi localization and spermatogenesis (Bentson et al., 2013; Chen et al., 2020; Dumin et al., 2006; Lehti et al., 2017; Mancini et al., 2000). The Golgin family includes GOLGA1 (Golgin-97), GOLGA2 (GM130), GOLGA3 (MEA-2), GOLGA4 (p230), and GOLGB1 (giantin) (Munro, 2011), with previous analysis showing that *Golga3*^repro27^ mice exhibited male-specific infertility which was associated with a nonsense mutation in the *Golga3* (Bentson et al., 2013). However, little is known about its phosphorylation during mouse spermatogenesis. In this study, a mouse model in which the serine at the S461 phosphorylation site was mutated to investigate whether mutations at phosphorylation sites affect spermatogenesis.

## Materials and Methods

### Animals

Mice were procured through the Experimental Animal Center of Nanjing Medical University, and subsequently randomly assigned to ventilated cages each housing four to five mice and were maintained in a standard facility under regulated conditions (20–22 °C; 50–70% humidity; 12-h/12-h dark/light exposure), with ad libitum water and food. The animals used in the experiments were over eight weeks old with mature male reproductive systems and were in good health. The mice were euthanized with CO_2_ upon study completion. This investigation received approval through the Institutional Animal Care and Use Committees of Nanjing Medical University (Approval No. IACUC-2004020-3), and carried out in line with relevant professional recommendations and the Guide for the Care and Use of Laboratory Animals.

### Mutation plasmid construction

Point mutant plasmids corresponding to the predicted GOLGA3 phosphorylation sites (Table 1) from the gnomAD database were constructed through designing forward and reverse primers (Table S1) to introduce the mutated base into the complete cDNA sequence leading to missense mutation of serine. PCR amplified complete cDNA encoding recombinant GOLGA3 which was then cloned into a pcDNA3.1 (+) vector (Thermo Fisher, Waltham, MA, USA) carrying a FLAG label. Next, the recombinant plasmid DNA was transformed into E.coli followed by extraction using the EndoFree Mini Plasmid Kit^®^ (DP118, Tiangen, Beijing, China) in accordance with the provided directions.

### Cell culture

The HEK293T and HeLa cellular cultures were procured through ATCC and expanded within DMEM together with high-glucose, 10% FBS (Gibco, USA), and penicillin / streptomycin (100 U/ml, Thermo Fisher, USA). Transfection was performed with Lipofectamine^®^ 2000 (11668019, Thermo Fisher, USA), following the provided protocol.

### *Golga3* mutation mouse generation

Cytosine base editors were used to generate *Golga3*^S461L/S461L^ mice using BE3 sgRNA for the introduction of the missense mutation. The sequences of the BE3 sgRNA and donor were 5′-AGACTCCCTTAGTTCTGAAGTGG-3′ and 5′GGTGGAACACAGCCACAGCAGCCAGCAGAAGCAAGACTTGCTTAGTTCTGAAGTGGACACTTTGAAGCAGTCTTGCTGGGATCTAGAGCGGGCCATGACT-3′respectively. After separate annealing and ligation of the cDNA sgRNA oligos onto BsaI-digested pUC57-T7-sgRNA vector, with sgRNA templates amplified by PCR using the Trans-PCR Forward (GAAATTAATACGACTCACTATAGG) and Reverse (AAAAGCACCGACTCGGTGCCA) primers, with the aid of MinElute PCR^®^ Purification Kit (28004, Qiagen, Germany). The sgRNA was prepared through MEGAshortscript^®^ (AM1354, Ambion, USA). Purification was performed with a MEGAclear Kit (AM1908, Ambion). After linearization of the BE3 plasmids (Addgene No. 73021) with AgeI and PmeI and purification as described above, BE3 mRNA was generated using a mMACHINE^®^ T7 Ultra Kit (AM1345, Ambion) with subsequent purification through RNeasy Mini^®^ (74104, Qiagen). This BE3 mRNA (50 ng/µL) was then used for inoculating mouse zygotes together with DonorDNA (50 ng/µL) and, BE3 sgRNA (20 ng/µL), before implantation in pseudo-pregnant mice. Tissue samples were collected from the toes of the seven-day-old offspring, followed by extraction of DNA from the tissue using Mouse Direct PCR^®^ (B40013, Biotool, USA). and amplified through primers (*Golga3*^S461L/S461L^ mice: F5′-ACCAGCCAGGATGTCTAT-3′, R5′-AGTAGGAAGGAGAGCAGAA-3′) and PrimeSTAR^®^ HS DNA Polymerase (DR010A, Takara, Japan), adopting the following PCR protocol, consisting of 95 °C (five minutes), followed by 35 cycles (95 °C for thirty seconds; 62 °C (−0.2°/cycle) for thirty seconds; 72 °C for thirty seconds) with a final step of 72 °C for five minutes. The products were sequenced by Sanger sequencing.

**Table 1:** Potential several missense mutations at the phosphorylation site of *GOLGA3*.

SNP	Location	Variant type	Alleles	gnom AD exomes
rs765540850	Chromosome 12:132804919	Missense variant	G/A	133381505
rs755956818	Chromosome 12:132786515	Missense variant	A/G	133363101
rs758140650	Chromosome 12:132807904	Missense variant	T/C	133384490
rs747449772	Chromosome 12:132807914	Missense variant	A/C	133384500
rs1161144811	Chromosome 12:132808254	Missense variant	G/A	133384840

### Dephosphorylation assays

The transfected cells containing the constructed mutant plasmids and plasmids with the full-length cDNA sequence were grown for 36 h and lysed using SDS Medicon RIPA lysis buffer without SDS containing 1x complete^®^ EDTA-free Protease Inhibitor Cocktail (Roche, Switzerland). After sonication, proteomic mixtures were placed on a rotary shaker for 45 min at a 20–30 rpm/min followed by high-speed centrifugation at 4 °C and measurement of protein concentrations in the supernatants. Mutant and normal proteins were placed into incubation, while exposed to calf intestinal alkaline phosphatase (CIP) (180 min; 37 °C water-bath); controls were not treated with CIP. 6 × loading buffer was introduced with dephosphorylated proteins and the samples underwent denaturing within a metal bath (95 °C; ten minutes).

### Western blotting

Cell lysates were prepared as above followed by electrophoresis of the proteins on SDS-PAGE, with consequent transport onto PVDF membranes. Blots underwent blocking (120 min; ambient temperature) using 5% skimmed milk within TBS-T (20 mM Tris, 150 mM NaCl, 0.1% Tween 20) before night-time incubation (4 °C) with anti-GOLGA3 (21193-1-AP, Proteintech, Waltham, MA, USA; 1: 5000) together with anti-p-CDK1(ab201008, Abcam, UK; 1: 5000). Following three TBS-T washing cycles, proteomic ontent samples were exposed (2 h, room temperature) to 2° antibodies (1:5000), evaluated visually through High-sig ECL^®^ Western Blotting Substrate (Tanon, China).

### Genotyping

Genomic DNA deriving through murine tail-tissue samples for the detection of mutations using PCR amplification and standard Sanger sequencing. The *Golga3*-edited mouse founders (F0) were identified using F-Primer 5′-ACCAGCCAGGATGTCTAT-3′ and R-Primer 5′-AGTAGGAAGGAGAGCAGAA-3′ together with PCR amplification (Fast-Taq Master Mix^®^, Vazyme, China). Amplicon was sub-cloned into the pMD19-T plasmid (TaKaRa, Wuhan, China), sequenced by Sanger sequencing. Founders showing base mutations at amino acid 461 of *Golga3* were crossed through WT C57BL/6 murines (3–4 generations) for circumventing off-targeted editing and resulting in the production of pure heterozygous animals. Sanger DNA sequencing of Golga3^S461L/S461L^ mice was carried out, with SnapGene (version 1.1.3) was used to plot the results. Each mouse represented a unique label, including genotype, sex, and birth date.

### Fertility test

The fertility of male mice was assessed through crossing male *Golga3*^S461L/S461L^ mice with female WT C57BL/6 mice; male *Golga3*^+/+^ mice were used as controls. The formation of vaginal plugs in the female mice was checked each morning. Information about each litter was recorded including birth date and the number of pups. The experimental unit requires an adult wild type mouse and mutant male mouse each in eight weeks. All experiments were performed in triplicate.

### Analysis of epididymal sperm

Sperm from *Golga3*^S461L/S461L^ and WT mice were assessed through computer-aided sperm analysis (CASA). Parameters for caudal epididymal sperm were determined, especially sperm quantity/ quality. After extrusion, samples were placed within human tubal-fluid culturing medium (In Vitro Care, Frederick, USA) and maintained at fixed temperature (37 °C) through a themostatic water bath. Sperm motility, progressive motility together with quantitative analysis for 10-µl aliquots of the sperm suspensions were assessed by CASA (Hamilton-Thorne Research, Beverly, MA, USA). An experimental unit requires an adult wild type mouse and mutant male mouse each in eight weeks. Experiments were performed in triplicate for ensuring statistical analysis robustness.

### Histologicy

Testis/epididymal tissue samples were collected through at least three *Golga3*^S461L/S461L^ murines, together with three *Golga3*^+/+^ male murines. Tissue samples were fixed overnight within Davidson’s fluid (MDF) and placed in 70% ethanol, followed by dehydration in an 80–90–100% ethanol gradient, paraffin-embedding, and sectioning (5-µm). Epididymal tissue underwent hematoxylin and eosin (HE) staining, while testis tissue underwent periodic acid Schiff (PAS) staining (395B, Sigma-Aldrich, USA). Smears of cauda epididymal sperm were also made, undergoing fixing with 4% paraformaldehyde within PBS (thirty minutes), rinsing, and HE staining.

### Immunofluorescence analysis

Paraffin sections of testicular tissue from wild-type and *Golga3*^S461L/S461L^ mice were deparaffinized and rehydrated. Antigen retrieval of sperm smears and slides of rehydrated testis tissue was undertaken by boiling samples within sodium citrate buffer (10 minutes). The slides were cooled, treated with 3-fold PBS wash-cycles (5 minutes/cycle), followed by blocking through 1% BSA within PBS (120 min at ambient temperature) prior to probing through 1° antibodies (night-time; 4 °C) (Table S2). Following this step, segments were subjected to 3 × PBST wash-cycles (0.05% Tween 20 within 1 × PBS) followed by assessment through 2° antibodies (Table S3) (120 min; ambient temperature). Nuclei were counterstained through Hoechst 33342 (Invitrogen, Waltham, MA, USA) (five minutes). After mounting with glycerin, segments were imaged through LSM800^®^ confocal fluorescence microscopy (Carl Zeiss AG, Jena, Germany).

### TUNEL assay

Quantitative analyses for apoptotic cells required TUNEL BrightRed^®^ Apoptosis Detection Kit (Vazyme, China), following the provided directions. After incubation with the BrightRed Labeling Buffer mixture, whereby wild-type and *Golga3*^S461L/S461L^ murine samples were treated to 3 × PBS wash-cycles (5 min each) in the dark,, stained with Hoechst 33342 (5 min, room temperature), mounted with glycerin, and examined and imaged under fluorescence microscopy as above.

### Statistical analysis

At least three replicates per experiment were performed. Datasets reflected mean ± the standard error. Cohorts were compared through one-way ANOVAs and unpaired two-tailed t-tests. *P* < 0.05 was deemed to confer statistical significance. The gray values of Western blot bands and mean fluorescence intensities were analyzed by Image J software. Microsoft Excel^®^ and GraphPad Prism^®^ 6.0 were employed for all such evaluations.

## Results

### Golga3 is conserved in various species and mutation of its phosphorylation site leads to abnormal localization of GOLGA3 in cells

**Figure 1:**
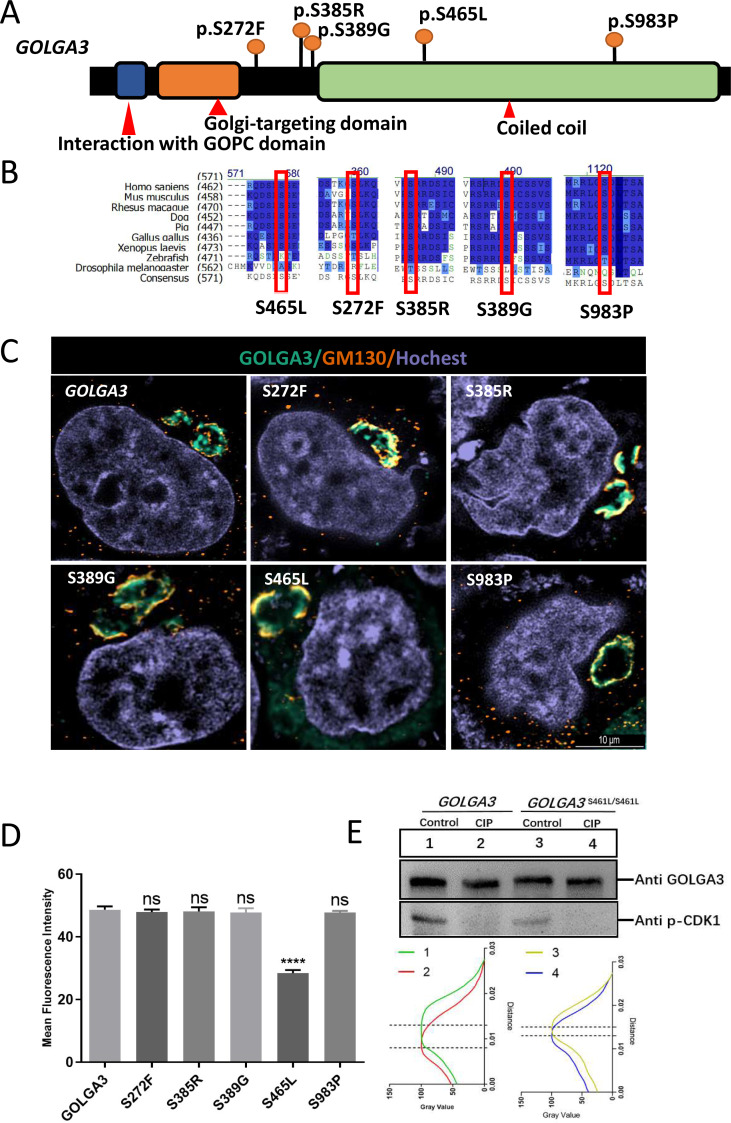
Conservation and mutation of phosphorylation sites leading to abnormal localization of GOLGA3. (A) The protein structure of GOLGA3 and its mutated phosphorylation sites (S272F, S385R, S389G, S465L, S983P). (B) Alignment of GOLGA3 protein sequences from different species, and an enlarged view of the mutant sites (red box). A dark blue background indicates residue identity while light blue indicates replacement with residues of similar properties. (C) Fluorescent detection of GOLGA3 (gray-green), GM130 (dark orange) and Hoechst (gray-blue) in HeLa cells transfected with wild-type and mutant plasmids. Scale bars = 10 µm. (D) Quantitative analysis of gray values for single-channel mean fluorescence intensity of GOLGA3. (E) GOLGA3 and GOLGA3^*S*461*L*/*S*461*L*^ proteins treated with calf intestinal alkaline phosphatase (CIP) or left untreated (Control), respectively, along with western blots (above) and quantification of bands’ gray values (below).

The GOLGA3 protein consists of a coiled-coil domain in the C-terminal region and two reported functional domains (domains for interaction with GOPC and Golgi-targeting) in the N-terminal region (http://www.uniprot.org/uniprot/Q08378) (Fig. 1A). Protein sequence alignment showed that *Golga3* was conserved in human, mouse, rhesus monkey, *Xenopus laevis* and zebrafish (Fig. 1B). Seven phosphorylation sites have been identified in the GOLGA3 protein and a further phosphorylation site inferred based on sequence similarity and mass spectrometry data (Bian et al., 2014; Olsen et al., 2010; Zhou et al., 2013). Mutations in the human *GOGLA3* gene were searched using the Ensembl database (https://asia.ensembl.org/Homo_sapiens/Gene/Variation_Gene), specifying missense mutations as a filtering criterion to obtain the frequency of such mutations at phosphorylation sites in the human gene corresponding with those in the mouse *Golga3* gene. By combining this information with that of conserved phosphorylation sites, five potential phosphorylation sites (Table 1; Figs. 1A and 1B) were identified. Constructed mutant plasmids with a flag tag were then transfected into HeLa cells prior to immunofluorescence co-localization staining with GM130 protein. It was found that GOLGA3^S465L^ showed reduced localization to the Golgi apparatus, with part of the protein dispersed within the cytoplasm. In contrast, GOLGA3^S272F^, GOLGA3^S385R^, GOLGA3^S389G^ and GOLGA3^S983P^ showed similar signals to the wild-type GOLGA3 protein (Fig. 1C). The fluorescence intensity of the GOLGA3 protein was subsequently quantified by analyzing gray values and statistical analysis of the mean fluorescence intensity in single channels, confirming the reduced localization of the protein to the Golgi apparatus (Fig. 1D). The S465 phosphorylation site in the human sequence corresponded to the S461 phosphorylation site in mouse. This site is located in the coiled coil domain and is strongly conserved in different species. To verify whether S461 was indeed phosphorylated, we transfected the constructed mutant plasmids and control plasmids into HEK293T cells. The mutant and normal proteins were then treated with CIP or were left untreated (Control) before western blotting. Quantification of the gray values of the protein bands showed a reduced band shift in the dephosphorylated GOLGA3^S465L^, confirming that S461 was phosphorylated (Fig. 1E).

### *Golga3*^**S461L/S461L**^ mice have normal fertility and normal spermatozoa

**Figure 2:**
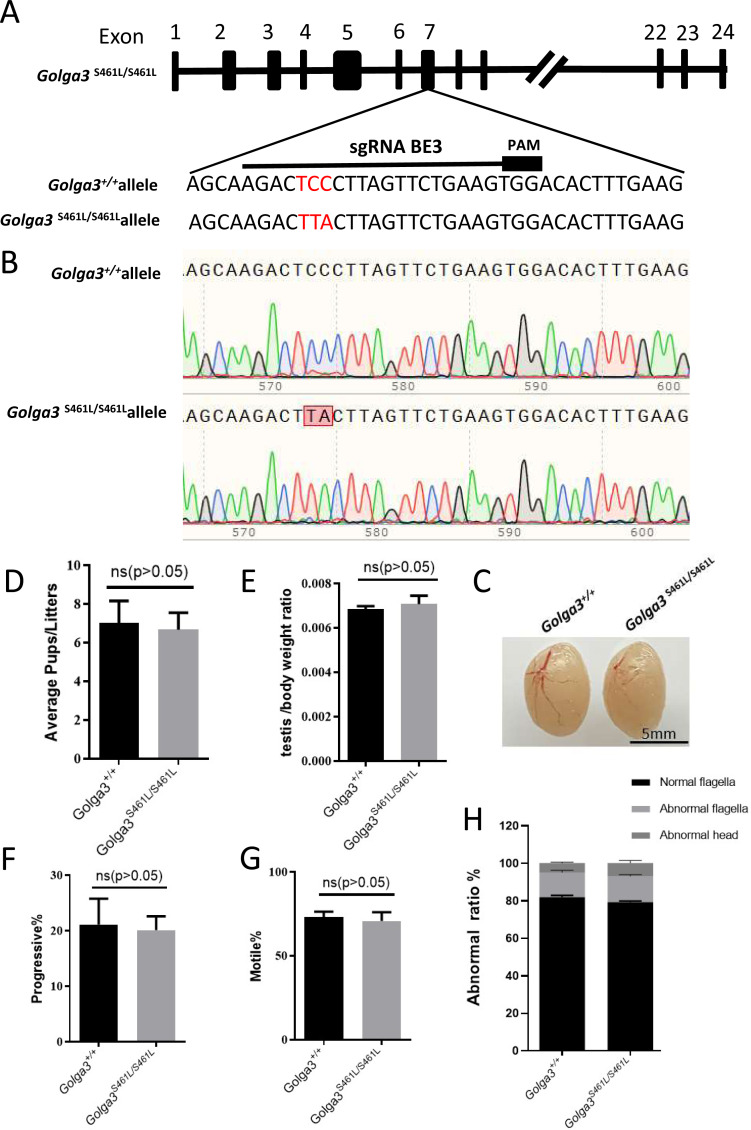
Generation of mutant mice and assessment of *Golga3*^*S*461*L*/*S*461*L*^ male mice fertility. (A) Schematic diagram of BE3-mediated *Golga3* editing; The sgRNA was designed based on exon 7 of *Golga3*; (B) The murine model of the S461L mutation was obtained *via* a CC-to-TA mutation, resulting in a missense mutation and was detected in *Golga3*^*S*461*L*/*S*461*L*^ mice by Sanger sequencing ; (C) Testes from wild-type and *Golga3*^*S*461*L*/*S*461*L*^ adult mice; (D) Average pups per litter in wild-type and *Golga3*^*S*461*L*/*S*461*L*^ mice, *n* = 3, *P* > 0.05, mean = 7 and 6.6667, 95% confidence interval: −4.367 to 3.701; (E) Average testis weight/body weight, *n* = 3, *P* > 0.05, mean =0.0068 and 0.0071, 95% confidence interval: −0.0009077 to 0.001374. (F) Progressive sperm from wild-type and *Golga3*^*S*461*L*/*S*461*L*^ mice, *n* = 3, *P* > 0.05, mean = 21.13 and 19.17, 95% confidence interval: −16.06 to 12.13; (G) Sperm motility in wild-type and *Golga3*^*S*461*L*/*S*461*L*^ mice, *n* = 3, *P* > 0.05, mean = 73.10 and 71.07, 95% confidence interval: −18.32 to 14.26; (H) Abnormal epididymal sperm counts from wild-type and *Golga3*^*S*461*L*/*S*461*L*^ mice, *n* = 3, *P* > 0.05.

To investigate the effects of mutation of the GOLGA3 phosphorylation site in the testes of C57BL/6 mice, a missense mutation was generated at S461 in exon 7 of the *Golga3* gene, in super-ovulated fertilized eggs using cytosine base editors (Fig. 2A). Compared with wild-type mice, the two cytosine bases responsible for encoding the serine at position 461 were simultaneously converted to thymine and adenine, respectively, in *Golga3*^S461L/S461L^ mice, and was confirmed by both Sanger sequencing and PCR genotyping (Fig. 2B).

It was found that male *Golga3*^S461L/S461L^ mice nevertheless exhibited normal development, including being viable and fertile. The testis morphology was also normal in adult *Golga3*^S461L/S461L^ mice, with no significant changes in the testis weight of adult *Golga3*^S461L/S461L^ mice (*N* = 3). The *p*-values for the average number of pups per litter as well as the average testis weight/body weight were 0.8298 and 0.6005, respectively, determined by t-tests performed with GraphPad Prism 8 software (Figs. 2C, 2D and 2E). Computer-assisted sperm analysis (CASA) was then used to evaluate semen quality of adult *Golga3*^S461L/S461L^ mice, including parameters such as the sperm motility ratio and progressive sperm ratio. In this case, the *p*-values for the latter two parameters were 0.7456 and 0.7182 respectively (Figs. 2F and 2G). In particular, the ratio of normal sperm in male *Golga3*^S461L/S461L^ mice was found to be similar to that of the control group, with no significant changes also noted in progressive motility (Fig. 2H).

### *Golga3*^**S461L/S461L**^ mice exhibited normal spermatogenesis

**Figure 3:**
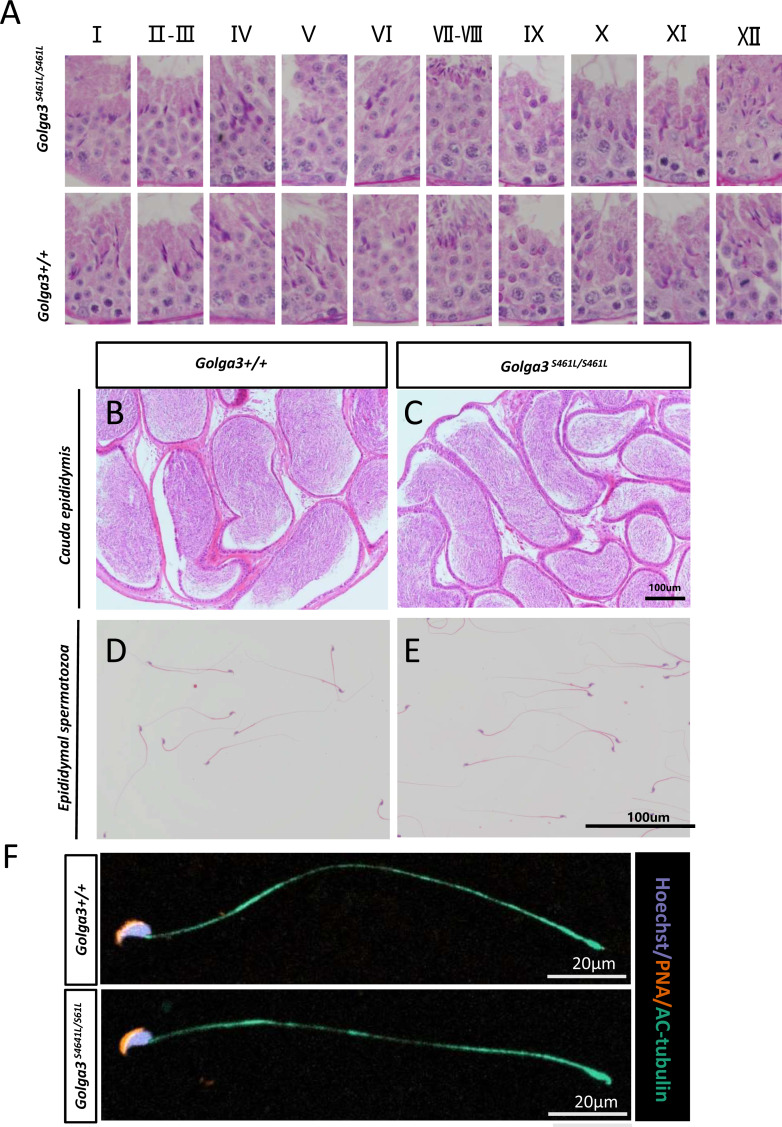
Normal spermatogenesis in *Golga3*^*S*461*L*/*S*461*L*^ mice. (A) Periodic Acid Schiff (PAS) staining of testicular sections from wild-type and *Golga3*^*S*461*L*/*S*461*L*^ mice. The epithelial cycle was divided into 12 stages recognized by PAS, according to changes in the acrosome and nuclear morphology of spermatids. Scale bar: 20 µm; HE staining of the cauda epididymis obtained from (B) wild-type and (C) *Golga3*^*S*461*L*/*S*461*L*^ mice. Scale bar: 100 µm; HE stained spermatozoa from (D) wild-type and (E) *Golga3*^*S*461*L*/*S*461*L*^ mice. (F) Fluorescent detection of AC-tubulin (gray-green) and PNA (dark orange) from wild-type and *Golga3*^*S*461*L*/*S*461*L*^ spermatozoa. Scale bars = 20 µm.

**Figure 4:**
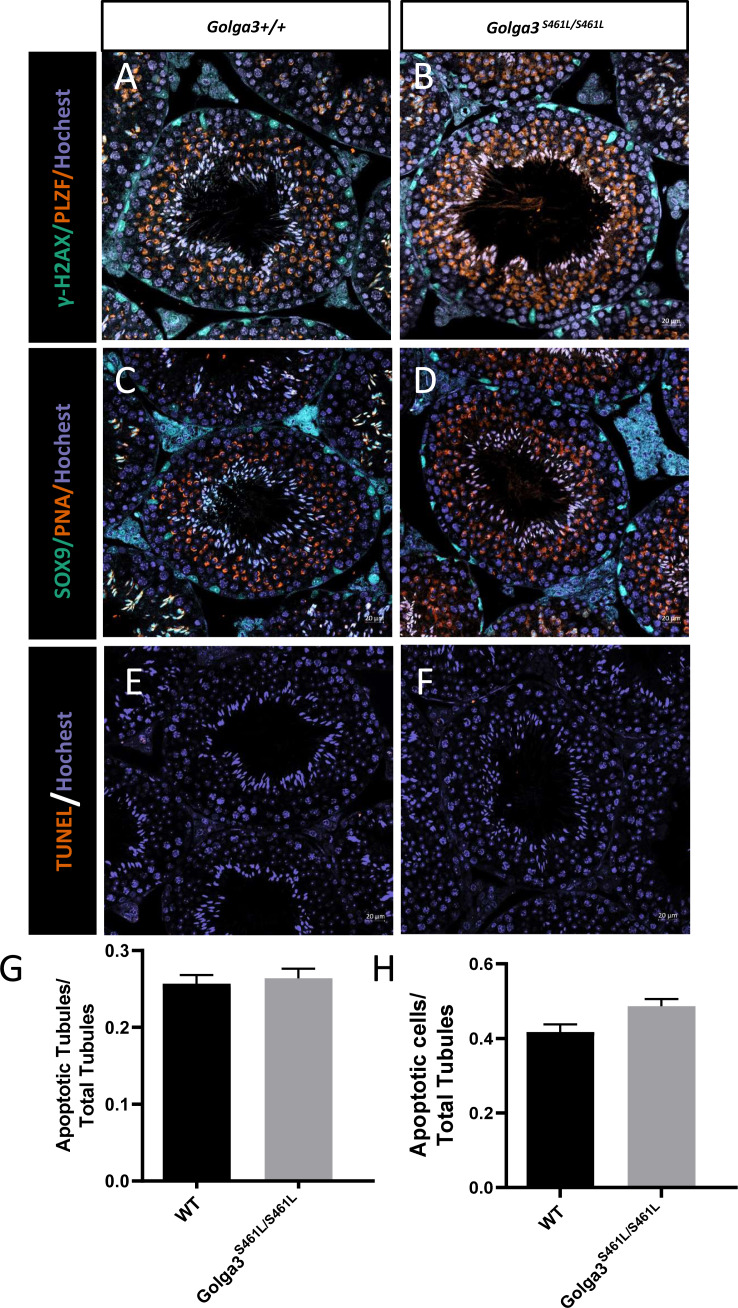
Spermatogenic markers appear normal and numbers of apoptotic cells are not significantly higher in *Golga3*^*S*461*L*/*S*461*L*^ mice. The spermatocytes (*γ*-H2AX) and spermatogonia (PLZF) were comparable in testis sections from both (A) wild-type and (B) *Golga3*^*S*461*L*/*S*461*L*^ mice; Spermatids (PNA) and Sertoli cells (Sox9) were similar in testis sections from both (C) wild-type and (D) *Golga3*^*S*461*L*/*S*461*L*^ mice. TUNEL analysis of (E) wild-type and (F) *Golga3*^*S*461*L*/*S*461*L*^ testicular sections; (G) Average number of apoptotic cells per seminiferous tubule; (H) Average number of apoptotic cells per seminiferous tubules, *n* = 3, *P* > 0.05. Scale bars = 20 µm.

Normal spermatogenic cells were observed in the PAS-stained seminiferous tubules of male adult *Golga3*^S461L/S461L^. Furthermore, histological analysis of each phase of spermatogenic tubule revealed the presence of post-meiotic round spermatids in the seminiferous tubules of adult *Golga3*^S461L/S461L^ mice in contrast to the *Golga3*^+/+^ mice (Fig. 3A). Normal numbers of spermatids and sperm were also observed in H&E-stained samples from these edited mice (Figs. 3B, 3C, 3D and 3E). Furthermore, no significant differences in sperm morphology were observed between the adult *Golga3*^S461L/S461L^ mice and wildtype mice based on the immunofluorescence staining of sperm smears (Fig. 3F). PLZF, *γ*-H2AX, PNA and Sox9 fluorescence signals were used to specifically identify proliferating spermatogonia, spermatocytes, spermatids, and Sertoli cells, respectively. The fluorescence images showed normal spermatogenesis in *Golga3*^S461L/S461L^ mice (Figs. 4A, 4B, 4C and 4D). Furthermore, to investigate the apoptosis of germ cells in spermatogenic tubules, TUNEL analysis of testicular sections was also performed. While sporadic dark orange fluorescence signals, indicative of apoptotic cells, were visible in the seminiferous tubules, these signals did not differ significantly between the *Golga3*^S461L/S461L^ and wild-type mice(Figs. 4E and 4F). Further *t*-test analysis showed that the *p*-values for the number of apoptotic cells/total tubules as well as the number of tubules/total tubules were 0.5070 and 0.0132, respectively (*P* > 0.05) indicating that there were no significant differences between the mutated and wild-type mice in terms of apoptosis in the seminiferous tubule (Figs. 4G and 4H, Table S4).

### Immunofluorescence co-localization and GOLGA3 protein levels are normal in *Golga3*^**S461L/S461L**^ mice

**Figure 5:**
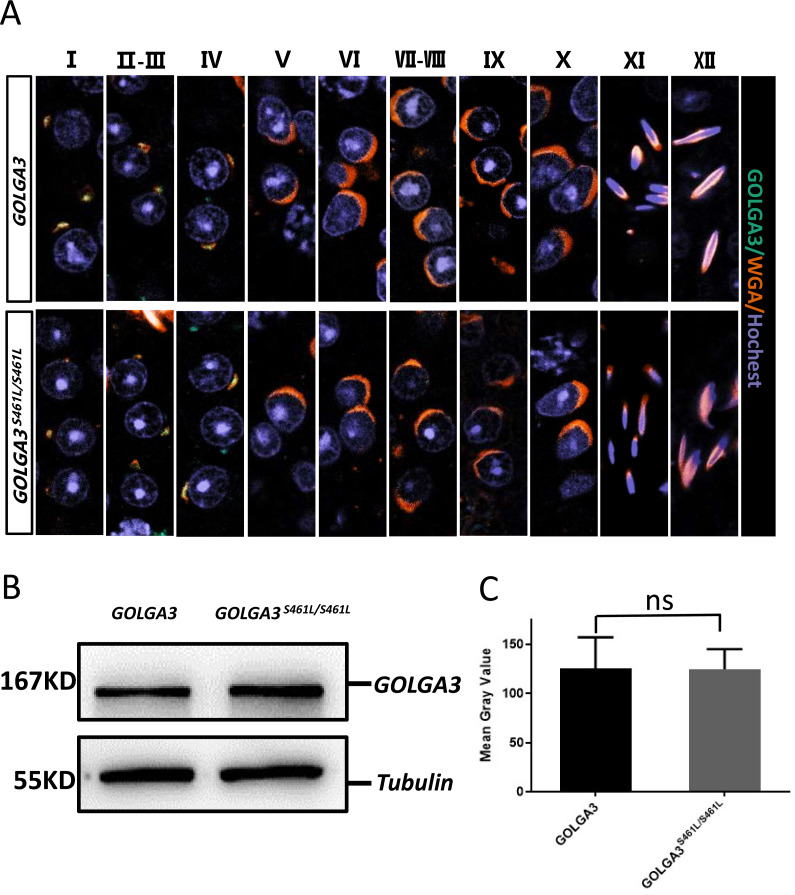
Normal immunofluorescence co-localization, along with protein levels of GOLGA3 in *Golga3*^*S*461*L*/*S*461*L*^ mice. (A) Spermatogenic phase of testicular seminiferous tubule section from GOLGA3 and *GOLGA3*^*S*461*L*/*S*461*L*^ mice, GOLGA3 (gray-green), WGA (dark orange) and Hochest (gray-blue). Scale bar: 20 µm. (B) Western blotting (left) and (C) analysis of gray band values (right).

During the early stages of spermatogenesis, the Golgi vesicles fuse to form sperm proacrosomal vesicles. The GOLGA3^S461L^ protein showed reduced Golgi localization, with some protein dispersed within the cytoplasm. To better understand the location of the GOLGA3 protein in *Golga3*^S461L/S461L^ mice, immunofluorescence co-localization was performed on mouse testicular sections. It was found that during stages 1–4 of spermatogenesis, GOLGA3 co-localized with Wheat Germ Agglutinin (WGA) (Fig. 5A), although there were no significant differences in fluorescence localization between mutant mice and wild type mice. Finally, to explore whether the S461L mutation affected the level of protein expression, western blotting and quantification of the protein bands were undertaken. This showed that the size of the GOLGA3^S465L^ band was similar to that of the wild-type, with no significant differences in the gray values between the two (Figs. 5B–5C).

## Discussion

Post translational modifications (PTMs) can occur shortly after translation or at any stage of a protein’s life cycle, with such changes helping to regulate protein folding, stability, cell localization, activity, or its interaction with other proteins or biomolecular species. As an important post-translational modification, protein phosphorylation affects protein activity by adding phosphate groups to protein kinases or phosphotransferases. This type of protein modification is widespread in all domains of life, including eukaryotes, bacteria and archaea (Macek et al., 2019). For instance, by applying quantitative and high-resolution mass spectrometry technology to the identification and quantification of phosphorylation sites, a comprehensive eukaryotic and prokaryotic phosphorylation proteome database has been preliminarily established (http://www.phosida.com). This database demonstrates that the levels of protein phosphorylation in *Homo sapiens*, *Mus musculus*, *Drosophila melanogaster,* and *Caenorhabditis elegans* are 35.00% (8,283/23,669), 39.01% (9,234/23,669), 10.05% (2,379/23,669), and 10.02% (2,373/23,669), respectively. On the other hand, phosphorylation levels tend to be low in prokaryote species (Gnad, Gunawardena & Mann, 2011). Thus, different groups of organisms show significant differences in the degree of post-translational protein phosphorylation, with mammals showing significantly higher levels of protein phosphorylation compared with other organisms. However, it remains to be established whether these phosphorylation sites play decisive roles in protein function.

Many critical steps in the spermatogenesis process involve protein phosphorylation or dephosphorylation, and as such, they require coordinated actions of kinases and phosphatases (Urner & Sakkas, 2003). Among these, histone phosphorylation is one of the most important post-translational modifications as it plays an important role in chromosome condensation as well as chromatin remodeling during mitosis/meiosis in spermatogenesis. Consequently, it is not surprising that blocking phosphorylation sites has also been reported to cause defects in the conversion of histone protamine, with increased retention of histones H3 and H4 in spermatozoa subsequently leading to impaired male fertility (Jha, Tripurani & Johnson, 2017). Before fertilization, sperm cells undergo capacitation to acquire the ability to fertilize the egg, and in this case, the activation of protein tyrosine phosphorylation is a key event in capacitation (Alvau et al., 2016). In addition to sperm maturation and sperm capacitation, phosphorylation has been reported to be associated with sperm motility. Indeed, phosphorylation of SEPT12 was previously shown to regulate the assembly of septin complexes in the sperm annulus. At the same time, mutation of S198E, the phosphorylation site of SEPT12, in the human protein (corresponding to S196E of SEPT12 in mouse) resulted in loss of sperm motility as well as the complete disappearance of the annulus/septin ring, ultimately leading to male infertility (Shen et al., 2017). Proteomic analyses have identified a number of phosphorylation sites, but studies on their functional significance remain scarce (Dempsey et al., 2021; Floyd, Drew & Marcotte, 2021; Gou et al., 2020). Indeed, much of the current research still remains focused on the function of upstream kinases that perform the phosphorylation processes. Functional studies of those kinases have only been performed for phenotypic analysis by generating knockout mice through gene-editing techniques. However, there is limited information on the functional role of specific phosphorylation sites (Crapster et al., 2020; Zhao et al., 2021). For this purpose, researchers are required to perform progressively larger numbers of experiments at later stages to demonstrate which downstream phosphorylation sites are truly involved in the phosphorylation or dephosphorylation processes.

As previously reported, the loss of germ cells and obvious vacuolation leads to testis atrophy in *Golga3* KO mice. At the same time, lower sperm concentrations and motility in the caudal epididymis were found to lead to male infertility in *Golga3* KO mice (Bentson et al., 2013). Nonsense mutations were found to result in the premature termination of translation, thereby indirectly knocking out GOLGA3. In contrast, missense mutations at phosphorylation sites do not prevent translation and allow the expression of GOLGA3 *in vivo*. Thus, while the knockout of GOLGA3 leads to a loss of protein functions and inhibits its biological role in spermatogenesis to damage reproductive ability, the mutation of a single phosphorylation site may instead only affect the activation of downstream kinases, resulting in a series of chain reactions. It should, however, be noted that not all mutated phosphorylation sites affect protein phosphorylation as this type of protein modification tends to jointly involve multiple phosphorylation sites (Cohen, 2000; Wang et al., 2016). This indicates that there is mutual synergy, antagonism, or complementation between phosphorylation sites. For instance, the S217 and S221 phosphorylation sites in MKK1 are involved in the protein’s regulatory functions. Phosphorylation of the two sites occurs synergistically, with the phosphorylation of one serine stimulating the rate of phosphorylation of the other even though the phosphorylation of a single site plays only a minor role (Chatterjee et al., 2021). The consequences of different missense mutations of the same phosphorylation site may also be different, as single mutations of phosphorylation sites may not necessarily have an effect if they are not biologically functional. For example, the S222, S225, and S235 phosphorylation sites on the NS5A protein are not involved in changes in translation and the resultant expression of the protein. Hence, the mutation of these sites has no apparent effect on protein function. In contrast, mutation of the S229A and S238A phosphorylation sites prevented NS5A translational downregulation, whereas the S229D and S238D mutations had no effect. More interestingly, the mutation of the S232D phosphorylation site directly inhibited the translational downregulation of NS5A (Kandangwa & Liu, 2019).

Analysis of the gnomAD database and immunofluorescence co-localization at the cellular level led to the preferential identification of S461 from eight other GOLGA3 phosphorylation sites in mice (http://www.uniprot.org/uniprot/P55937). The GOLGA3^S465L^ protein showed weakened Golgi localization with scattered protein visible in cytoplasm. However, missense mutation of the S461 (corresponding to the S465 phosphorylation site in the human protein) did not affect the function of the entire protein in mouse spermatogenesis. Although the S461L mutation did not affect spermatogenesis, it should be remembered that fluorescence co-localization of other phosphorylation sites of the GOLGA3 protein was only performed *in vitro* at the cellular level. No mutant mouse model was generated to verify whether these changes affected spermatogenesis *in vivo*. Therefore, the results do not indicate whether male fertility is preserved in mice with mutations in other phosphorylation sites or whether mutations in multiple superimposed phosphorylation sites can have an effect on mouse spermatogenesis. While further research is warranted to address these questions, this study nevertheless provides a reference for subsequent studies related to protein phosphorylation while being helpful in elucidating the molecular mechanisms of male infertility.

## Conclusions

*Golga3* is a highly conserved gene in the testis, and its encoded protein is involved in spermatogenesis especially in apoptosis signaling and in Golgi complex-mediated effects. Although infertility was observed in *Golga3* KO male mice and even though GOLGA3^S465L^ showed weakened Golgi localization with protein visible in cytoplasm, this abnormal localization of GOLGA3 did not adversely affect fertility or spermatogenesis in male C57BL/6 mice. Therefore, mutation at the S461 GOLGA3 phosphorylation site does not affect mouse spermatogenesis.

##  Supplemental Information

10.7717/peerj.15133/supp-1 Table S1 Primers used for construction of mutant plasmids and verification of *GOLGA3* Mutations

10.7717/peerj.15133/supp-2 Table S2 List of antibodies

10.7717/peerj.15133/supp-3 Table S3 List of antibodies

10.7717/peerj.15133/supp-4 Table S4 TUNEL-positive apoptotic cells and tubules count

10.7717/peerj.15133/supp-5 Figure S1 Figure 1 raw data PZFX is a Prism file form and can be opened using GraphPad Prism 5.0 or later. We used GraphPad Prism 6.0.

10.7717/peerj.15133/supp-6 Figure S2 Figure 2 raw data PZFX is a Prism file form and can be opened using GraphPad Prism 5.0 or later. We used GraphPad Prism 6.0.

10.7717/peerj.15133/supp-7 Figure S3 Figure 3 raw data PZFX is a Prism file form and can be opened using GraphPad Prism 5.0 or later. We used GraphPad Prism 6.0.

10.7717/peerj.15133/supp-8 Figure S4 Figure 4 raw data PZFX is a Prism file form and can be opened using GraphPad Prism 5.0 or later. We used GraphPad Prism 6.0.

10.7717/peerj.15133/supp-9 Figure S5 Figure 5 raw data PZFX is a Prism file form and can be opened using GraphPad Prism 5.0 or later. We used GraphPad Prism 6.0.

10.7717/peerj.15133/supp-10 Supplemental Information 10 Raw Data: Blots

10.7717/peerj.15133/supp-11 Supplemental Information 11 Author checklist

10.7717/peerj.15133/supp-12 Supplemental Information 12 GOLGA3 protein level-WB

## Additional Information and Declarations

### Data Availability

The following information was supplied regarding data availability: The raw data is available in the Supplemental Files.
