# Supplementary material for: Mutation of S461, in the GOLGA3 phosphorylation site, does not affect mouse spermatogenesis
Source: PeerJ. 2023 Apr 17;11:e15133. doi: 10.7717/peerj.15133 (PMC10117384; doi:10.7717/peerj.15133)
Supplement: Table S1 [file peerj-11-15133-s001.docx]

**Supplementary materials:**

**Table S1. Primers Used for Construction of mutant plasmids and Verification of *GOLGA3* Mutations**

| **Primer Names** | **Primer Sequences(5`-3`)** |
| --- | --- |
| **GOL** |  |
| GOLGA3-For | accagccaggatgtctat |
| GOLGA3-Rev | agtaggaaggagagcagaa |
| GOLGA3-272-For | attctaccaagggttccctgaagcagaacagaagcagtg |
| GOLGA3-272-Rev | ctgttctgcttcagggaacccttggtagaatcgggagc |
| GOLGA3-385-For | cggggaggtgcggagtcggagagacagcatctgcag |
| GOLGA3-385-Rev | gatgctgtctctccgactccgcacctccccg |
| GOLGA3-389-For | cggagtcggagagacagcatctgcagcagcgtgt |
| GOLGA3-389-Rev | cgctgctgcagatgctgtctctccgactccgcacc |
| GOLGA3-465-For | agcagcggcaggattcgctgagctcggaggtggac |
| GOLGA3-465-Rev | acctccgagctcagcgaatcctgccgctgctgg |
| GOLGA3-983-For | atgaggcggctgggctcagacttgaccagcgccca |
| GOLGA3-983-Rev | cgctggtcaagtctgagcccagccgcctcatc |
| 3xFlag-Golga3-F1 | aaggatgacgatgacaagcttgatggagcatcagccaaacagga |
| 3xFlag-Golga3-R1 | cacttcagaactaagggagtcttgcttctgctggctgc |
| 3xFlag-Golga3-F2 | cagcagaagcaagactcccttagttctgaagtggacactttgaagc |
| 3xFlag-Golga3-R2 | ttcttggcctcttgttgcagct |
| 3xFlag-Golga3-F3 | agctgcaacaagaggccaagaa |
| 3xFlag-Golga3-R3 | ccacactggactagtggatcctcattggcccagcccgt |
