# Supplementary material for: Mutation of S461, in the GOLGA3 phosphorylation site, does not affect mouse spermatogenesis
Source: PeerJ. 2023 Apr 17;11:e15133. doi: 10.7717/peerj.15133 (PMC10117384; doi:10.7717/peerj.15133)
Supplement: Table S2 [file peerj-11-15133-s002.docx]

**Supplementary materials:**

**Table S2. List of antibodies**

| **Antibodies** | | **SOURCE** | **IDENTIFIER** |
| --- | --- | --- | --- |
| **GOL** |  | |  |
| Rabbit polyclonal anti-SOX9 | Millipore, Billerica, MA, USA | | Millipore, Billerica, MA, USA |
| Rabbit polyclonal anti-gamma H2A.X | Abcam, Cambridge, MA, USA | | Cat#ab2893 |
| Goat polyclonal anti-PLZF | RnD Systems, Minneapolis, MN,USA | | Cat#AF2944 |
| Mouse monoclonal anti-Acetylated Tubulin | Sigma, St.Louis, MO, USA | | Cat#T7451 |
| Rabbit polyclonal anti-GM130 | ABclonal | | A5344 |
| Mouse polyclonal anti-Flag | MBL | | PM020 |
| Rabbit polyclonal anti-GOLGA3 | proteintech | | Cat#21193-1-AP |
