# Supplementary material for: Mutation of S461, in the GOLGA3 phosphorylation site, does not affect mouse spermatogenesis
Source: PeerJ. 2023 Apr 17;11:e15133. doi: 10.7717/peerj.15133 (PMC10117384; doi:10.7717/peerj.15133)
Supplement: Table S3 [file peerj-11-15133-s003.docx]

**Supplementary materials:**

**Table S3. List of antibodies**

| **Antibodies** | | **SOURCE** | **IDENTIFIER** |
| --- | --- | --- | --- |
| **GOL** | Invitrogen, Carlsbad, CA, USA | |  |
| Donkey anti-Mouse IgG, Alexa Fluor 488 | Invitrogen, Carlsbad, CA, USA | | Cat#A-21202 |
| Donkey anti-Rabbit IgG, Alexa Fluor 488 | Invitrogen, Carlsbad, CA, USA | | Cat#A-21206 |
| Donkey anti-Mouse IgG, Alexa Fluor 555 | Invitrogen, Carlsbad, CA, USA | | Cat#A-31570 |
| Donkey anti-Rabbit IgG, Alexa Fluor 555 | Invitrogen, Carlsbad, CA, USA | | Cat#A-31572 |
