# Supplementary material for: Mutation of S461, in the GOLGA3 phosphorylation site, does not affect mouse spermatogenesis
Source: PeerJ. 2023 Apr 17;11:e15133. doi: 10.7717/peerj.15133 (PMC10117384; doi:10.7717/peerj.15133)
Supplement: Table S4 [file peerj-11-15133-s004.docx]

**Supplementary materials:**

**Table S4. TUNEL-positive apoptotic cells and tubules count**

|  | | **Total Tubules** | | **Apoptotic cells** | **Apoptotic Tubules** | **Apoptotic Tubules/**  **Total Tubules** | **Apoptotic cells/**  **Total Tubules** |
| --- | --- | --- | --- | --- | --- | --- | --- |
|  |  | | |  |  |  |  |
| WT-1 | | | 100 | 40 | 25 | 0.250 | 0.400 |
| WT-2 | | | 100 | 44 | 27 | 0.270 | 0.440 |
| WT-3 | | | 100 | 41 | 25 | 0.250 | 0.410 |
| Mut-1 | | | 120 | 57 | 33 | 0.275 | 0.475 |
| Mut-2 | | | 120 | 61 | 32 | 0.267 | 0.508 |
| Mut-3 | | | 120 | 57 | 30 | 0.250 | 0.475 |

**TUNEL, TdT-mediated dUTP Nick-End Labeling.**
