# Supplementary material for: Mutation of S461, in the GOLGA3 phosphorylation site, does not affect mouse spermatogenesis
Source: PeerJ. 2023 Apr 17;11:e15133. doi: 10.7717/peerj.15133 (PMC10117384; doi:10.7717/peerj.15133)
Supplement: Figure S1 — PZFX is a Prism file form and can be opened using GraphPad Prism 5.0 or later. We used GraphPad Prism 6.0. [file peerj-11-15133-s005.zip › Fig1_Raw_data/figure1-Raw data/Multispecies conservation analysis.pdf]

Section 1

```
                           (1)  1     10        20        30        40             57
Homo sapiens               (1)  ---------------------------------------------------------
Mus musculus               (1)  ---------------------------------------------------------
Rhesus macaque             (1)  ---------------------------------------------------------
Dog                        (1)  ---------------------------------------------------------
Pig                        (1)  ---------------------------------------------------------
Gallus gallus              (1)  ---------------------------------------------------------
Xenopus laevis             (1)  ---------------------------------------------------------
Zebrafish                  (1)  ---------------------------------------------------------
Drosophila melanogaster    (1)  MDTRSWRKVLFQWIGECHFIESNYITLEQSDLDSFFSVFIQKIQETETVKGKNELQD
Consensus                  (1)
```

Section 2

```
                          (58)  58          70        80        90        100          114
Homo sapiens               (1)  --------------MDGASAEQDGLQEDRSHSGPSSLPEAPLKPPG--------PLV
Mus musculus               (1)  --------------MDGASAKQDGLWESKSSSDVSSCPEASLETVG-------SLAR
Rhesus macaque             (1)  --------------MDGASAEQDGLQEDRSHSGPLALPEGPLKPPGPVVPPDQDPLV
Dog                        (1)  -----------------MDRMPAGSSSGTPAH-----PEALPKPPN--------SLV
Pig                        (1)  --------------MDGVVAMQDGPEDRSSNG-----PEALPKPPG--------SLA
Gallus gallus              (1)  --------------MESSSVQQDVLLDNRSSSG---APGTSQEHGDCN-----ANSA
Xenopus laevis             (1)  -------------------MHLTPMTMEQQSSPRILLCDSLNEERSTVSKVETFDAK
Zebrafish                  (1)  -----------------MENGNNGMVSIEAKPPQHPDAQHCKNPNSN------TVES
Drosophila melanogaster   (58)  QPTEQSPLVQEFLAHNYPEFIAQQDDKEVDLPLDCLYVYTLLLHYSCVKKPSLFFHN
Consensus                 (58)                MD     QDGL E  S S     PEA  P
```

Section 3

```
                         (115)  115  120       130       140       150       160       171
Homo sapiens              (36)  PPDQQDKVQCAEVNRASTEGESP--DGPGQGGLCQNGPTPPFPDPPSSLDPTTSPVG
Mus musculus              (37)  LPDQQDTAQDASVEVNRGFKEEGSPDRSSQVAICQNG---QIPDLQLSLDPTTSPVG
Rhesus macaque            (44)  PPDQQDKAQRAEVNRASTEGESP--DGPGQGGLCQNGPMPPSPDPPSSLDPTTSPVG
Dog                       (28)  PPNQWDKAQCAGIEVHRASDRDGDLDVPSKVNTCQNGPMLPIPDPSSSLGTPTSPVG
Pig                       (31)  LLDQQEEAQCTNVEVNRASDEGG-----SQGDTCQNGPMPQFPDPPS-------SVG
Gallus gallus             (36)  LPATGDEVNTSSVNLSEVPNEEGSLELSSKADACQNGPELLFPDSPISFDPTSSPQG
Xenopus laevis            (39)  TASIAEGIRVTSINSSHLPKEVDNVLISSKEDACANGPPLHGTEPISPVDSTSNVPT
Zebrafish                 (35)  LKPERSLVTEKIPNGHVKAELLPNGEAMVEGQDVGGGSTPPSATPSPGSRLSDSSPS
Drosophila melanogaster  (115)  ICNKLPELTQTCIASFFRETVDRLLTREYLSQAIANVAVVYRQGVSTSVSPCLPSSS
Consensus                (115)  LP Q D VQ A VN      E    D  S G  CQNGP    PDP SSLDPTTSPVG
```

Section 4

```
                         (172)  172    180       190       200       210            228
Homo sapiens              (91)  PDASPGVAGFHDNLRKSQGTSAEGSVRKEALQSLRLSLPMQETQLCSTDSPLPLEKE
Mus musculus              (91)  PDASTGVDGFHDNLRNSQGTSAEGSVRKEALQSLRLSLPMQETQLCSTASSLPLEKE
Rhesus macaque            (99)  PDASPGVAGFHDNLRKAQGTSAEGSVRKEALQSLRLSLPMQETQLCSTDSSLPLEKE
Dog                       (85)  PDASPGVAGFHDNLRKSQGTSAEGSVRKEALQSLRLSLPMQETQLCSIEASLPLEKE
Pig                       (76)  PDASPGVAGFHDNLRKSQGTSAEGSGRKEALQSLRLSLPMQETQLCSTEASLPLEKE
Gallus gallus             (93)  QESSP----------------------------------------GSVESSLPLENE
Xenopus laevis            (96)  ISASPGLAGFSDNLMESSANG--IVLQKEALQSLKLSLHIQETDLCFSEAPLSLERE
Zebrafish                 (92)  TELDPGVVSFPDSLERSEGS----VHTGDALQSLRLSIPMQETELSSTDPCVEMENE
Drosophila melanogaster  (172)  LSPDPRSDDAPCPSTPSSSSS------QPSSSTPQLRNHREQLRLNGCDMPPPSTPK
Consensus                (172)  PDASPGVAGFHDNLRKSQGTSAEGSVRKEALQSLRLSLPMQETQLCSTDSSLPLEKE
```

Section 5

```
                         (229)  229        240       250       260       270            285
Homo sapiens             (148)  EQVRLQARKWLEEQLKQYRVKRQQERSS--QPATKTRLFSTLDPELMLNPENLPRAS
Mus musculus             (148)  EQVRLQARKRLEEQLMQYRVKRHRERSS--QPATKMKLFSTLDPELMLNPENLPRAS
Rhesus macaque           (156)  EQVRLQARKWLEEQLKQYRVKRQQERSS--QPATKTRLSSTLDPELMLNPENLPRAS
Dog                      (142)  ELVRLQARKRLEEQLKQYRVKRQQERSS--QPAARMRLSSTLDPELMLNPEALPRAS
Pig                      (133)  EQVRLQARKRLEEQLKQYRVKRQQERSS--QPVTKTRLFSTLDPELMLNPETLPRAS
Gallus gallus            (110)  EQIRLQARRRLEEQLRQYRVKRHQERSN--QSTSKSRPSSTLDPELMLNPEILPRAS
Xenopus laevis           (151)  EQIRLQARRRLEEQLKQYRVKRHQERSN--HSTPKSRPSSTLDPELMFNPETLPRAS
Zebrafish                (145)  EKIRQEARRRLEEQLKQYRAQRHTERTR--RSTPKSRPFSTLDPELMLHPEGLPRAN
Drosophila melanogaster  (223)  TELLEQRTKELRGIRTQLEVVRYEKALLEEQQTEKDELIKVLNKEKMMAKMELEKLR
Consensus                (229)  EQVRLQARKRLEEQLKQYRVKRHQERSS  QP TKSRL STLDPELMLNPE LPRAS
```

Section 6

```
                          (286)  286                 300       310       320       330        342
Homo sapiens              (203)  TLAMTKEYSFLRTSVPRGPKVGSLGLPAH-PREKKTSKSSK----IRSLADYRTEDS
Mus musculus              (203)  TVAVTKEYSFLRTSVPRGPKVGSLGLLAH-SKEKKNSKSSK----IRSLADYRTEDP
Rhesus macaque            (211)  TVAMTKEYSFLRTSVPRGPKVGSLGLPAH-PKEKKSSKSSK----IRSLADYRTEDS
Dog                       (197)  AVALTKEYSFLRTSVPRGPKVGSLGLLTH-PKGKKSSKSSK----IRSLADYKTEDV
Pig                       (188)  AVAMTKEYSFLRTSVPRGPKVGSLGLLAH-PKEKKSSKSSK----IRSLADYRTEES
Gallus gallus             (165)  TVAMTKEYSFLRTSVPRGPKLGSLGLPAS-SKERKSSKSSKSS-KIRSLADYRTEDS
Xenopus laevis            (206)  TVSMTKEYTFLRTNVPRGPKLGSLGLPSS-SKEKKSSRSSKSSNKIRSLADYRTEEL
Zebrafish                 (200)  TVAMTKEYSFLRTSVPRGPKLGSLGIPSSREKKSKSSRSSK----IHSLADYKTPES
Drosophila melanogaster   (280)  NVKLTEEHHDNESHHIMPYEFEHMKGCLLKEIGLKESLIAEITDKLHDLRVENSELS
Consensus                 (286)  TVAMTKEYSFLRTSVPRGPKVGSLGLPAH  KEKKSSKSSK    IRSLADYRTEDS
```

Section 7

```
                          (343)  343    350             360       370       380                399
Homo sapiens              (255)  NAGNSGGNVPAPDSTKGSLKQNRSSAASVVSEISLSPDTDDRLENTSLAGDSVSEVD
Mus musculus              (255)  SDS--GGLGSTADAVGSSLKQSRSS-TSVVSEVSPSSETDNRVESASMTGDSVSEAD
Rhesus macaque            (263)  NAGNSGGNVPVPDSTKGSLKQNRSSAASVVSEISLSPDTDDRLENASLAGDSVSEVD
Dog                       (249)  SAGSSGGDAAAVDSARVSLKQNRSSVVSVVSELSLGPEADDHLEN----VDNMSEAD
Pig                       (240)  DAQNSGGSVPAVDSARASLKQNRSSGTSVVSEVSLCPEADDRLENASLTGDNVSEAD
Gallus gallus             (220)  GSGSSAGNLMATDLPGGTLKQSRSGPASVVSEISLPFDTDDRIENSSLAGDSISEID
Xenopus laevis            (262)  EYKN--SSPGYSESSSGSLKPSRGSMTSVISEIG--MDSSEHFDGSSFGGDNGSELD
Zebrafish                 (253)  EGS--GGAVSASADSSFTSLHSTISSVSTVSEISISSEANNHSESSQLIRDNISEVD
Drosophila melanogaster   (337)  EKLNLAGKRLLEYTDRIRFLESRVDDLTRIVSSRDVMISSLESDKQELDKCLKEARD
Consensus                 (343)   A NSGG V A DS RGSLKQSRSS  SVVSEISL  DTDDRLENASL GD VSEVD
```

Section 8

```
                          (400)  400       410       420       430       440             456
Homo sapiens              (312)  G---------NDSDSSSYSSASTRGTYGILSKTVGTQDTPYMVNGQEIPADTLGQFP
Mus musculus              (309)  G---------NESDSSSHSSLSARGACGVLGN-VGMPGTAYMVDGQEISAEALGQFP
Rhesus macaque            (320)  G---------NDSDSSSYSSASTRGTYGVLSKTVGTQDTPYMVNGQEIPADTLGQFP
Dog                       (302)  G---------NESDSSSHSSVSARGTCGLLMNTLGTQEASYVVNGQEIAAGALGQFP
Pig                       (297)  G---------NESDSSSYSSVSTRGALSLLANSLGTREAPYVVSGQEVAAAALGQFP
Gallus gallus             (277)  GSEAGMRLDGNESDSSTYSSVSGKGLCNNLPNSEGKERIPYTINGQKIHPDAIGQFP
Xenopus laevis            (315)  GSEIGIRQDGNESDSSTYSSVSTN-RGVYAQATTPKQEADYSVNGQVIAAEDVGHYP
Zebrafish                 (308)  GSESGFRADGNDSDSSSYSSVSTTGTYNMLSAIVNRPKAPYTVEGREIAAEAMGHFP
Drosophila melanogaster   (394)  DLHNRIEVLNASSDLLDCSLSPNTTPENLASSVIDKQLREKEHENAELKEKLLNLNN
Consensus                 (400)  G         NESDSSSYSSVSTRG   LLS TVG QE PYMVNGQEIAADALGQFP
```

Section 9

```
                          (457)  457          470       480       490       500          513
Homo sapiens              (360)  SIKDVLQAAAAEHQDQGQEVNGE----VRSRRDSICSSVSLESSAAETQEEMLQVLK
Mus musculus              (356)  SIKDVLQAAAAQHQDQNQEANGE----VRSRRDSICSSVSMESSLAEPQDELLQILK
Rhesus macaque            (368)  SIKDVLQAAAAEHQDQGQEVNGE----VRSRRESICSSVSLESSAAETQEEMLQVLK
Dog                       (350)  SITDVLQAAAAEHQDRGQEVNGE----ARSRTDSMCSSISLESSAAETHDAMLQALK
Pig                       (345)  SISDVLQAAAAEHQDQRQEVNGE----TRSRADSICSSVSMESSVAETHDEMLQVLK
Gallus gallus             (334)  SISEVLQAAAVEHQAQGQEFNGE----IRSRRDSISSSVSMESSVAGTHDEMLQVLK
Xenopus laevis            (371)  SLSEVLQAAATECIALEQEINGE----PRSRRDSISSSISIESSIAGTHDELLQVLK
Zebrafish                 (365)  SLQEVLQAATEERHMEELEQDREGSVEPRSRRDSFSSSVSYGSSVMGTHDEMLQVLK
Drosophila melanogaster   (451)  SQRELCQALSSFLQKHNIDHEFP---VEWTSSSLLSTISAIESKFVNTLEKSTQMKK
Consensus                 (457)  SI DVLQAAAAEHQDQ QEVNGE     RSRRDSICSSVSLESSVAETHDEMLQVLK
```

Section 10

```
                          (514)  514   520       530       540       550       560       570
Homo sapiens              (413)  EKMRLEGQLEALSLEASQALKEKAELQAQLAALSTKLQAQVECSHSSQQ--------
Mus musculus              (409)  DKRRLEGQVEALSLEASQALQEKAELQAQLAALSTRLQAQVEHSHSSQQ--------
Rhesus macaque            (421)  EKMRLEGQLEALSLEASQALKEKAELQAQLAAVSTKLQAQVECSHSSQQ--------
Dog                       (403)  DKMRLEGQVEALSAEASQALKEKAELQAQLAALNTRLQAQVEHSHNSQQ--------
Pig                       (398)  EKMRLEGQLEALSLEASQALKEKAELQAQLAALSTRLQAQVEHSHNSQQ--------
Gallus gallus             (387)  EKMRLEGQLEALSAEANQALKEKAELQAQLAALNMKLQAQVEHSQSSQQ--------
Xenopus laevis            (424)  EKMRLEGQLESLTSEASQALKEKTELQAKLAAVSTKLQAELENQEASQK--------
Zebrafish                 (422)  EKMRLEGQLESLSSEASQALKEKTELQAQLATVNAQLKAQVEQTQAGQE--------
Drosophila melanogaster   (505)  ECDVQSVCVEKLLEKCKLLSVSLGCQPKELDGFEATIPEAMESGFESSRECETILSC
Consensus                 (514)  EKMRLEGQLEALS EASQALKEKAELQAQLAALSTKLQAQVE SHSSQQ
```

Section 11

```
                        571      580       590       600       610             627
                 (571)
Homo sapiens       (462) ---RQDSLSSEVDTLKQSCWDLERAMTDLQNMLEAKNASLASSNNDLQVAEEQYQRL
Mus musculus       (458) ---KQDSLSSEVDTLKQSCWDLGRAMTDLQSMLEAKNASLASSNNDLQVAEEQYQRL
Rhesus macaque     (470) ---RQDSLSSEVDTLKQSCWDLERAMTDLQNMLEAKNASLASSNNDLQVAEEQYQRL
Dog                (452) ---KQDSLSSEVDTLKQSCWDLERAMNDLQNMLEAKNASLASSNSDLQVAEEQYQRL
Pig                (447) ---RQDSLSSEVDTLKQSCWDLEQAMADLQNMLEAKNASLASSNSDLQVAEEQYHRL
Gallus gallus      (436) ---KQESLSSEVATLKQSCWDLERAMADLQNTLEAKNASLASSNNDLQLAEEQYQRL
Xenopus laevis     (473) ---KQASLSSEMLNLKKSCLDLERAMADLQNSLEVKNSSLSSLGNDLQVAEEQYQRL
Zebrafish          (471) ---RQSTLKTEVTTLRSNCSALEKAMVDLQTNLEGKNASLASLGNDLEVAEEQYQRL
Drosophila melanogaster (562) CHMKVVDIASKNNDLELDNERLNDKCAELKSIIDRGDQHLADINLQLIEKEKQIKDV
Consensus          (571)    KQDSLSSEVDTLKQSCWDLERAM DLQNMLEAKNASLASSNNDLQVAEEQYQRL
```

Section 12

```
                        628         640       650       660       670           684
                 (628)
Homo sapiens       (516) MAKVEDMQRSMLSKDNTVHDLRQQMTALQSQLQQVQLERTTLTSKLKASQAEISSLQ
Mus musculus       (512) MAKVEDMQRNILSKDNTVHDLRQQMTALQSQLQQVQLERTTLTSKLQASQAEITSLQ
Rhesus macaque     (524) VAKVEDMQKSMLSKDNTVHDLRQQMTALQSQLQQVQLERATLTSKLKASQAEISSLQ
Dog                (506) MAKVEEMQRNILSKDNTVHDLRQQMTALQNQLQQVQLERTTLSSKLKASQAEISSLQ
Pig                (501) MAKVEEMQRSMLSKDNTVHDLRQQMTALQSQLRQVQLERTALSSKLQASQAEISSLQ
Gallus gallus      (490) MLKVEDMQKSVLTRDSTVHDLRQQLASLQSQLQKVQLERTTLTNKLKASETEITSLQ
Xenopus laevis     (527) MGKVEELQRIIIQKDNAVHDLRQQTVSLQYQLQQVQLDRSTLTNKLKASKTEIASLQ
Zebrafish          (525) MVKVEEMQQSLNAKDNTVSELRQQMGGLQTQLQRVQSERNALQSRLKTSQAEVDSLQ
Drosophila melanogaster (619) GAEIQELRKRNINLENMLSQIADKEASAASHAQHLKQCGELLRAKYEVCRNELIAKN
Consensus          (628) MAKVEEMQRSILSKDNTVHDLRQQMTALQSQLQQVQLERTTLTSKLKASQAEISSLQ
```

Section 13

```
                        685  690       700       710       720       730         741
                 (685)
Homo sapiens       (573) SVRQWYQQQLALAQEARVR-LQGEMAHIQVGQMTQAGLLEHLKLENVSLSQQLTETQ
Mus musculus       (569) HARQWYQQQLTLAQEARVR-LQGEMAHIQVGQMTQAGLLEHLKLENVSLSHQLTETQ
Rhesus macaque     (581) SVRQWYQQQLALAQEARVR-LQGEMAHIQVGQMTQAGLLEHLKLENVSLSQQLTETQ
Dog                (563) HVRQWYQQQLALAQEARVR-LQGEMAHIQVGQMTQAGLLEHLKLENVSLSHQLTETQ
Pig                (558) SVRQWYQQQLALAQEARVR-LQGEMAHIQVGQMTQAGLLEHLKLENVSLSHQLTETQ
Gallus gallus      (547) NVRQWYQQQLVLAQEARVR-LQSEMANIQAGQMTQAGVLEHLKLENVALSQQLTETQ
Xenopus laevis     (584) QTRDWYQQQLTLAQGARVR-LQSEMANIQAGQMSQAGVLEHLKIENVTLSHQLTETQ
Zebrafish          (582) QLRLWYQQQLNLAQEARVR-LQGEMANMQAGQMTQFGVLENLKIENVTLSHKLTETN
Drosophila melanogaster (676) AAQDELVRMMMVPDGETLNGRVRQLIDLEMMHDEHNKMYAQMLRQLNELSAKHDNMT
Consensus          (685)  VRQWYQQQL LAQEARVR LQGEMAHIQVGQMTQAGLLEHLKLENVSLSHQLTETQ
```

Section 14

```
                        742     750       760       770       780             798
                 (742)
Homo sapiens       (629) HRSMKEKGRIAAQLQGIEADMLDQEAAFMQIQEAKTMVEEDLQRRLEEFEGER----
Mus musculus       (625) HRSIKEKERIAVQLQSIEADMLDQEAAFVQIREAKTMVEEDLQRRLEEFEGER----
Rhesus macaque     (637) HRSMKEKGRIAAQLQGIEADMLDQEAAFLQIQEAKTMVEEDLQRRLEEFEGER----
Dog                (619) HRSIKEKERIAVQLQGIEADMLDQEAAFMQIQEAKTMVEQDLQRRLEEFEDEK----
Pig                (614) QRSIKEKERIAAQLQGIEADMLDQEAAFVQIQEAKTMVEEDLQRRLEEFEDEK----
Gallus gallus      (603) HRSIKEKERIAAQLQNIEADMLDQEAAFMQIQEAKTMVEEDLQRKLEEFEDEK----
Xenopus laevis     (640) HKSLKEKERIATQLQNIEADMLDQEAAFHQIQEAKSMVEEDLQRKLEEFEQEK----
Zebrafish          (638) HRSIKEKERIAVQLQSIEADMLTQEAAIHQIQEAKTMVEEDLQHKLEEFEEER----
Drosophila melanogaster (733) HSHLDFVKRTEIELETKNAQIMAFDEHNNHFDRFLTRIFTLLRSRNCPKSTTMGSAT
Consensus          (742) HRSIKEKERIA QLQ IEADMLDQEAAFMQIQEAKTMVEEDLQRRLEEFE EK
```

Section 15

```
                        799       810       820       830       840             855
                 (799)
Homo sapiens       (682) --ERLQRMADSAASLEQQLEQVKLTLLQRDQQLEALQQEHLDLMKQLTLTQEALQSR
Mus musculus       (678) --EQLQKVADAAASLEQQLEQVKLTLFQRDQQLAALQQEHLDVIKQLTSTQEALQAK
Rhesus macaque     (690) --ERLQRMADSAASLEQQLEQVKLTLLQRDQQLEALQQEHLDLMKQLTLTQEALQSR
Dog                (672) --EQLQKMAALAVSLEQQLEQVNLTLQQRDQQLEVLQQEHLDLLRQLTSTQETLQTR
Pig                (667) --EQLQKMVASVATLEQQLEQVKLTLHQRDQQLEALQQEHLDLLKQFTSTQENLQAR
Gallus gallus      (656) --EQLQKMADSAATLEQELDQVKLTLHQRDLQLESLQQEHLDLMKQFTMTQEMLHTK
Xenopus laevis     (693) --EQLQKLADSATALELELEQVKLNLHQRDLQLEALQQEHLEILKQLSVTQEILHTK
Zebrafish          (691) --EHLLKLANTATTLERELEQVKLVLFQKDSQLESLQREHLELMKQLTTTQETLQTK
Drosophila melanogaster (790) NFLESMHIEKRFENIEMLIEGQLLSADDLKRELDDLRSKNEELAKQNINGIIKRN--
Consensus          (799)   EQLQKMADSAASLEQQLEQVKLTL QRDQQLEALQQEHLDLMKQLT TQE LQTK
```

```
                                                         Section 16
                          (856) 856            870       880       890       900         912
           Homo sapiens   (737) EQSLDALQTHYDELQARLGELQGEAASREDTICLLQNEKIILEAALQAAKSGKEELD
           Mus musculus   (733) GQSLDDLHTRYDELQARLEELQREADSREDAIHFLQNEKIVLEVALQSAKSDKEELD
        Rhesus macaque    (745) EQSLDALQTHYDELQARLGELQGEAASREDTICLLQNEKIILEAALQAAKSGKEEFD
                    Dog   (727) EQSLGDLQVHYDELHARLEELQGEAASRDDTIRFLQNEKIVLEVALQAARSGREELD
                    Pig   (722) EQALGDLRVRCDELQARLDELQGEAASRDDTICVLQNEKIVLEVALQAAR--REELD
          Gallus gallus   (711) EQTLDDLQTQYDELKARLEEFQSDATSKDDMIQYLQNEKIVLEVALQTAKASQDQLD
         Xenopus laevis   (748) DQSLNDLQMRYDELEGRLLELQSDTASKDDTIDYLQNQKIVLEVALQAAKMEQDGLD
              Zebrafish   (746) EQALNQLEARYQELQVQLEELQTDASAKEETLQYLQNEKIVLEVALQAARADKSELD
Drosophila melanogaster   (845) -KFITSLEVNTEKVKQYITDLEEEAFKRKQKVVQLEN---TLSKEQSNAKEMAQRLD
              Consensus   (856) EQSL DLQ  YDELQARLEELQ EAASRDDTI YLQNEKIVLEVALQAAKS KEELD

                                                         Section 17
                          (913) 913    920       930       940       950               969
           Homo sapiens   (794) RGARRLEEGTEETSETLEKLREELAIKSGQVEHLQQE---TAALKKQMQKIKEQFLQ
           Mus musculus   (790) RGARRLEEDTEETSGLLEQLRQDLAVKSNQVEHLQQE---TATLRKQMQKVKEQFVQ
        Rhesus macaque    (802) RGARRLEEGTEETSETLEKLREELAIKSGQVEHLQQE---TATLKKQTQKIKEQFLQ
                    Dog   (784) RGAKRLEEGTEETSQILEQLRQELAVKSSQVEHLQQE---AATLKKQIQKIKEQFLQ
                    Pig   (777) PGSKRQEESTEETSETLERLREELAIKSSQVEHLQQE---AATLKKQTQKIKEQFLQ
          Gallus gallus   (768) EGTKRLGEDTEVTSEILEQLRQEMAIRSSQVENLQQE---NGSLKKQVQKVKEQFLQ
         Xenopus laevis   (805) HGVRNLQEGTVAAAEVLAKLRQELSIKSCQVETLQKD---NSTIKKQTQKVKEQFLQ
              Zebrafish   (803) EGAEKLGEEVLVASDTLDQLRQEVQVKATQIEALQHE---NGTLKKQAQKLKEQFMQ
Drosophila melanogaster   (898) IAQQEIKDYHVEAIRFINTIRDRLQQDFNGVNTPQQLGTCMTEFLKMYDQMEVRYEE
              Consensus   (913)  GARRLEE TEETSE LE LRQELAIKS QVEHLQQE    ATLKKQ QKIKEQFLQ

                                                         Section 18
                          (970) 970       980       990       1000      1010           1026
           Homo sapiens   (848) QKVMVEAYRRDATSKDQLISELKATRK-------------RLDSELKELRQELMQVH
           Mus musculus   (844) QKVMVEAYRRDATSKDQLINELKATKK-------------RLDSEMKELRQELIKLQ
        Rhesus macaque    (856) QKVMVEAYRRDATSKDQLISELKATRK-------------RLDSELKELRQELMQVH
                    Dog   (838) QKVMVEAYRRDATSKDQLIGELKATKK-------------RLDSELKELRQELIKLQ
                    Pig   (831) QKVMVEAYRRDAASKDQLVSELKATKK-------------RLDAEAKELRQELIRLQ
          Gallus gallus   (822) QKVMVEAYRRDASSKDQLISELKATKK-------------RLDSEMKELKRELLQIQ
         Xenopus laevis   (859) QKVMVEAYRRDASSKEQLISELKTTKK-------------KLDSEVKELRQKLLWLQ
              Zebrafish   (857) QKVMVEAYRRDASSKEQLISELKASKK-------------RLVAEVKDLKQELLKTE
Drosophila melanogaster   (955) SSSLVEKLTESQAKLEMQVAELQVELENKDTNQHSGALIKQLNDTIQNLEKVNAKLS
              Consensus   (970) QKVMVEAYRRDATSKDQLISELKATKK             RLDSELKELRQELIKLQ

                                                         Section 19
                         (1027) 1027         1040      1050      1060      1070         1083
           Homo sapiens   (892) GEKRTAEAELSRLHREVAQVRQHMADLEGHLQSAQKERDEMETHLQSLQFDKEQMVA
           Mus musculus   (888) GEKKTVEVEHSRLQKDMSLVHQQMAELEGHLQSVQKERDEMEIHLQSLKFDKEQMIA
        Rhesus macaque    (900) GEKRAAEAELSRLHREAAQVRQQMADLEGHLQSAQKERDEMETHLQSLQFDKEQMVA
                    Dog   (882) GEKKSTEVEHVRLQKEVSHIRQQMVDLEGHLQVVQRERNEMETHLQSLQFDKEQVVA
                    Pig   (875) GEKRSVEVEHARLQKDVTQARQQVADLEGHLQSVQRERDQMEMNLQSLQFDKEQMVA
          Gallus gallus   (866) VEKQSLETEHSKLQKEVTEVHQQMVEIENHLQSVQKERDEMETRLQSLQFDKEQMAS
         Xenopus laevis   (903) GEKSAAEVEQARVRKEMSQLQQQMEELESNFQAVQKERDDMESRLQSLQFDKDQVAT
              Zebrafish   (901) GEKKSAVQEQARLQKEVERVQQQMNGLEAHLQSVQTERDQLDSQLQSLQFDQNQLAA
Drosophila melanogaster  (1012) EDNTVSHTVHSKLNESLLKAQKELDLRAKIIENLEASERNLSMKLCELKDLKNKLKS
              Consensus  (1027) GEKKSAE EHSRLQKEVS V QQM DLEGHLQSVQKERDEMET LQSLQFDKEQMVA

                                                         Section 20
                         (1084) 1084  1090      1100      1110      1120      1130      1140
           Homo sapiens   (949) VTEANEALKKQIEELQQEARKAITEQKQK----MRRLGSDLTSAQKEMKTKHKAYEN
           Mus musculus   (945) LTEANETLKKQIEELQQEAKKAITEQKQK----MKRLGSDLTSAQKEMKTKHKAYEN
        Rhesus macaque    (957) VTEANEVLKKQIEELQQEARKAITEQKQK----MRRLGSDLTSAQKEMKTKHKAYEN
                    Dog   (939) LTEANQVLKKQIEELQQEATKAITEQKQR----MKRLGSDLSSAQKEMKTKHKAYEN
                    Pig   (932) LTEANEVLKKQIEELQQEATKAITEQKQK----MKRLGSDLSSAQKEMKTKHKAYEN
          Gallus gallus   (923) LAEANQTLKLQVEQMQEEAKKAITEQKQK----MKRLGSDLTSAQKEMKAKHKAYEN
         Xenopus laevis   (960) LAQDNAALKEQVELMQNEAKKAITEQKQR----MKRIGSDLTSAQKEMKIKHKAYEN
              Zebrafish   (958) VTEENENLRKRVEQMQNEARTAISEQKVK----MKRLGTDLTSAQKEMKAKHKAYEN
Drosophila melanogaster  (1069) SDEKIAQIKETYEEQIKALQAKCDMEAKKNEHLERNQNQSLTQLKEDALENCVLMST
              Consensus  (1084) LTEANE LKKQIEELQQEAKKAITEQKQK    MKRLGSDLTSAQKEMKTKHKAYEN
```

```
                                                                  Section 21
                         (1141) 1141     1150      1160      1170      1180            1197
         Homo sapiens (1002) AVGILSRRLQEALAAKEAADAELGQLRAQGGSSDSSLALHERIQALEAELQAVSHSK
          Mus musculus (998) AVSILSRRLQEALASKEATDAELNQLRAQSTGGSSDPVLHEKIRALEVELQNVGQSK
      Rhesus macaque (1010) AVGILSRRLQEALAAKEAADAELGQLRAQGGSGDSSLALHERIQALEAELQAVSHSK
                   Dog (992) AVGILSRRLQEALAAKESAESELSQLRAQVAGGGSNILLHERIQALEAELQAVSHSK
                   Pig (985) AVGILSRRLQEALAAKEAAEAELGQLRAQAASSSDDFPLHERIQALETELQTVGHSK
          Gallus gallus (976) AVSILSRRLQESLTAKESAEAELSKLKAQITDGGSNQIAQERIQALETELQAVRSSK
        Xenopus laevis (1013) AVGILSRRLQEALTAKETAEAELNKLKAQLSDVENNQAFQAKLQSLQNELQTVCHSK
           Zebrafish (1011) AVGILSRRLQEALAAKETAEAELDKLKAQVADGGNSQELQAKVKSLQGELQAVSQSK
Drosophila melanogaster (1126) KLEELQAKLQEGQQLVDSQKLELDMNRKELALVKSAYEAQTKLSDDLQRQKESGQQL
           Consensus (1141) AVGILSRRLQEALAAKEAAEAEL QLRAQLA G S    LHERIQALE ELQAV HSK
                                                                  Section 22
                         (1198) 1198       1210      1220      1230      1240          1254
         Homo sapiens (1059) TLLEKELQEVIALTSQELEESREKVLELEDELQ-ESRGFRKKIKRLEESNKK-----
          Mus musculus (1055) ILLEKELQEVITMTSQELEESREKVLELEDELQ-ESRGFRRKIKRLEESNKK-----
      Rhesus macaque (1067) TLLEKELQEVIALTSQELEESREKVLELEDELQ-ESRGFRKKIKRLEESNKK-----
                   Dog (1049) TMLEKELQEIITLTSQELEEHREKVLELEDELQ-ESRGFRRKIKHLEELNKK-----
                   Pig (1042) TMLERELQEVISMTSQELEEQREKVLELEDELQ-EARGFRRKIKHLEESNKK-----
          Gallus gallus (1033) LMLEKELQEVISLTSQELEEYREKVLELEDELQ-ESRGFRRKIKRLEEINKK-----
        Xenopus laevis (1070) AMLERELQEVISLTSQELEEYREKVIELEDELQ-EARGFRRKIKRLEELNKK-----
           Zebrafish (1068) AMLEKELQEVITLTSTELEEYQEKVLELEDEQLQESRNFKKRIRRLEDANKK-----
Drosophila melanogaster (1183) VDNLKVELEKERKELAHVNSAIGAQTKLSDDLECQKESGQQLVDNLKVELEKERKEL
           Consensus (1198)  MLEKELQEVISLTSQELEE REKVLELEDELQ ESRGFRRKIKRLEESNKK
                                                                  Section 23
                         (1255) 1255 1260      1270      1280      1290      1300      1311
         Homo sapiens (1110) --LALELEHEKGKLTGLGQSNAALREHNSILETALAKREADLVQLNLQVQAVLQRKE
          Mus musculus (1106) --LALELEHERGKLTGLGQSNAALREHNSILETALAKREADLVQLNLQVQAVLQRKE
      Rhesus macaque (1118) --LALELEHEKGKLTGLGQSNAALREHNSILETALAKREADLVHLNLQVFKLVKLSM
                   Dog (1100) --LALELEHERGKLTGLGQSNAALREHNSILETALAKREADLVQLNLQVQAVLQRKE
                   Pig (1093) --LALELEHERGKLTGLGQSNAALREHNSILETALAKREADLVQLNLQVQAVLQRKE
          Gallus gallus (1084) --LALELEHERGKLTGLSQSNAALREHNNVLETALAKREADLVQLNLQVQAVLKRKE
        Xenopus laevis (1121) --LTLEVEHEKGKLTGLGQSNAALREHSNILEAALAKRESDLVQLNLQVQAVLKRKE
           Zebrafish (1120) --LALELEHEKGKLSGLGKSHSALREHANILEAALAKREADLVQLNLQVQAVLKRKE
Drosophila melanogaster (1240) AQVKSVIEAQTKLSDDLQREKESAQQLVDNLKVELDKERKELAQVNSAFEAQTKLSD
           Consensus (1255)   LALELEHEKGKLTGLGQSNAALREHNSILETALAKREADLVQLNLQVQAVLKRKE
                                                                  Section 24
                         (1312) 1312     1320      1330      1340      1350              1368
         Homo sapiens (1165) EE---DRQMKHLVQALQASLEKEKEKVNSLKEQVAAAKVEAGHNRRHFK--------
          Mus musculus (1161) EE---DRQMKQLVQALQVSLEKEKMEVNSLKEQMAAARIEAGHNRRHFK--------
      Rhesus macaque (1173) AA---ARSTCRDGCLPQAAFPSRR---------------------------------
                   Dog (1155) EE---DRQTKQLVHTLQAALEREKAKVHSLKEQVAASKAEAGHNRRHFR--------
                   Pig (1148) EE---DRQMKQLVQALQVALQKEKVTVRSLEEQVAAAKAEAGHNRRHFK--------
          Gallus gallus (1139) EE---DQQMQQLIQALQASLEKEKSKVKDLKEQVAAAKADAAHNRRHYR--------
        Xenopus laevis (1176) EE---DRQMKELVQTLQDALQKEKTKVSSLTEQLAAAKAEAAHNRRHYR--------
           Zebrafish (1175) EE---DQQMRQLVQTLQTALEKEKIKVKDLTEQVAEAKLEAAHNRRHYR--------
Drosophila melanogaster (1297) DLQREKESAQQLVDNLKVELDKERKELAQVKSVIEAQTKLSDDLQRQKESAQQLVDN
           Consensus (1312) EE   DRQMKQLVQ LQ ALEKEK KV SLKEQVAAAK EAGHNRRHFR
                                                                  Section 25
                         (1369) 1369       1380      1390      1400      1410          1425
         Homo sapiens (1211) ------AASLELSEVKKELQAKEHLVQKLQAEADDLQIREGKHSQEIAQFQAELAEA
          Mus musculus (1207) ------AATLELSEVKKELQAKEHLVQTLQAEVDELQIQDGKHSQEIAQFQTELAEA
      Rhesus macaque (1194) -------------AGHAGGARQFPAFLFFYARIR---E--GKHSQEIAQFQAELAEA
                   Dog (1201) ------AATLELSEVKKELQAKEQLVQKLQAEAEGLQIQEEKHSQEIVQFQAELAEA
                   Pig (1194) ------AATLELSEVKKELQAKEQEVQRLQAEADGLQIQEGKHSQEIAQFQAELAEA
          Gallus gallus (1185) ------AAALELNEVKKELHAKELLVQALQAEVDKLQVEDEKHSQEVSQFQQELAEA
        Xenopus laevis (1222) ------AAALELSEIQKELQAKQQLIQALQTEAEKLQSQDKKNTQELSQFQQELAEA
           Zebrafish (1221) ------AAVLELSEIKKDLQAKEELVKALEKEATTLAAQDEKHSEEVSRFREELADA
Drosophila melanogaster (1354) LKVELDKERKELAKVKSVIEAQTKLSDDLQRQKESAQQLVDNLKVELDKERKELAQV
           Consensus (1369)       AA LELSEVKKELQAKE LVQ LQAEAD LQIQD KHSQEIAQFQ ELAEA
```

Section 26

```
                           (1426)  1426          1440      1450      1460      1470        1482
            Homo sapiens (1262)  RAQLQLLQKQLDEQLSKQPVGNQEMENLKWEVDQKER--------------------
            Mus musculus (1258)  RTQLQLLQKKLDEQMSQQPTGSQEMEDLKWELDQKER--------------------
         Rhesus macaque (1233)  RVQLQLLQKQLDEQLSKQPVGNQEMENLKWEVDQKER--------------------
                     Dog (1252)  RTQLQLLQKQLDEQLNKQPIGNQEMENLKWEVEQKER--------------------
                     Pig (1245)  RTQLQFLQKQLDEQLSKEPIGNQEMENLKWEVDQKER--------------------
            Gallus gallus (1236)  RSQLQLLQKKLDDKLSEQPLVSQEVEDLKWEVEQKER--------------------
          Xenopus laevis  (1273)  HSQLQLLQKELDEQMSKRPEISQEVEDLKWEMDQKEH--------------------
                Zebrafish (1272)  HTQLQILQKQLDDELNKQPVTNQEVEDLKWEVEQKQR--------------------
 Drosophila melanogaster (1411)  NSAFEAQTKLSDDLQRQKESAQQLVDNLKVELDKERKELAQVKSAIGAQTKLSDDLE
                Consensus (1426)  RTQLQLLQKQLDEQLSKQPVGNQEMENLKWEVDQKER
```

Section 27

```
                           (1483)  1483   1490      1500      1510      1520               1539
            Homo sapiens (1299)  ----EIQSLKQQLDLTEQQGRKELEGLQQLLQNVKSELEMAQEDLSMTQKDKFMLQA
            Mus musculus (1295)  ----EIQSLKQQLDLTEQQGKKELEGTQQTLQTIKSELEMVQEDLSETQKDKFMLQA
         Rhesus macaque (1270)  ----EIQSLKQQLDLTEQQGRKELEGLQQLLQNVKSELEMAQEDLSMTQKDKFMLQA
                     Dog (1289)  ----ELQSLKQQLNMTEQQSKKELDGIQQLLQNMKAELEMVREDLSLTQKDKFMLQA
                     Pig (1282)  ----EIQALKQQLDLTEQQSTKELEGVQQSLQNIKSELEMAREDLSMTQKDKFMLQA
            Gallus gallus (1273)  ----EIGTLKQQLDMSEQRSHKELEGMQVVLQNIKTELEVVREDLSVTQKDKFMLQA
          Xenopus laevis  (1310)  ----EIQAIRQQLDMTEQRNQRELEGVQILLQNLKLEFEAVRDDLTDAQKDKFMLQA
                Zebrafish (1309)  ----EMEAQKQQLEMVEQCHQREMQSQQDMLQTIKVELEMVQEELNGTRKDKFMLQA
 Drosophila melanogaster (1468)  CQKESVQQLVDNLKVELEKERKELAKVNSAFEAQTKLSDDLKLQKEDAQREVFLVKE
                Consensus (1483)      EIQSLKQQLDLTEQQ RKELEGVQQLLQNIKSELEMVREDLSMTQKDKFMLQA
```

Section 28

```
                           (1540)  1540      1550      1560      1570      1580          1596
            Homo sapiens (1352)  KVSELKNNMKTLLQQNQQLKLDLRRGAAKTRKE----------PKGEASSSNPATPI
            Mus musculus (1348)  KVSELKNNMKTLLQQNQQLKLDLRRGAAKK-KE----------PKGESNSSSPATPI
         Rhesus macaque (1323)  KVSELKNNMKTLLQQNQQLKLDLRRGAAKTRKE----------PKGEASSSNPATPI
                     Dog (1342)  KVSELKNNMKTLLQQNQQLRLDLRRGTTKMRKE----------LKGETSSSGPVTPV
                     Pig (1335)  KVSELKNNMKTLLQQNQQLKLDLRRGAAKTRKE----------LKEATSSS-PVTPV
            Gallus gallus (1326)  KVSELKNSMKSLLQQNQQLKMDLKHGKMKK-------------RKGENNSSNPVTPV
          Xenopus laevis  (1363)  KVSELKNSMKTLLQQNQQLKLDLRQGRIKKRKE----------LKSDATSSNPVTPV
                Zebrafish (1362)  KVGELRNSMKTVLQQNNQLKQDLKNSRLRK--Q----------RMDLKGESTPVTPV
 Drosophila melanogaster (1525)  RLVKEKREFEVKLATLEDIIETLEMRCTQMEEERATAYEQINKLENRCQEKDNVKSS
                Consensus (1540)  KVSELKNNMKTLLQQNQQLKLDLRRG  K RKE           KGE SSS PVTPV
```

Section 29

```
                           (1597)  1597         1610      1620      1630      1640         1653
            Homo sapiens (1399)  KIPDCPVPASLLEELLRPPPAVSKEPLKNLNSCLQQLKQEMDSLQRQMEEHALTVHE
            Mus musculus (1394)  KIPDCPVPASLLEELLRPPPAVSKEPLKNLNNCLQQLKQEMDSLQRQMEEHTITVHE
         Rhesus macaque (1370)  KIPDCPVPASLLEELLKPPPAVSKEPLKNLNSCLQQLKQEMDSLQRQMEEHTLTVHE
                     Dog (1389)  KIPDCPVPASLLEELLRPPPAVSKEPLKNLNSCLQQLKQEMDSLQRQMEAHTVTVHE
                     Pig (1381)  KIPDCPVPASLLEELLRPPPAVSKEPLKNLNSCLQQLKQEMDSLQRQMEAHTVTVHE
            Gallus gallus (1370)  KIPDCPVPAALLEELLKPSTAVSKEPLKNLNSCLRQLKQEMDSLQRQMEEHTITVHE
          Xenopus laevis  (1410)  KIPDCPVPASLLEELLKPTSANSKEPLNNLNNCLRQLKHEMDSLQRQMEAHTITVHE
                Zebrafish (1407)  KIPDCPVPASLLDELLKPSTSVNKEPLNNLHNCLKQLKQEMDSLQKQMEEHTVTVTS
 Drosophila melanogaster (1582)  QLQVETFKVECLHHQLKSEMATHNSLVEDLNRKLAENVSKLDFVQSRLMTEIAEHNQ
                Consensus (1597)  KIPDCPVPASLLEELLKPPPAVSKEPLKNLNSCLQQLKQEMDSLQRQMEEHTITVHE
```

Section 30

```
                           (1654)  1654  1660      1670      1680      1690      1700     1710
            Homo sapiens (1456)  SLSSWTPLEPATASPVPPGGHAGPRGDPQRHSQSRASKEGPGE--------------
            Mus musculus (1451)  SLSSWAQVEAAP------AEHAHPRGDTKLHNQNSVPRDGLGQ--------------
         Rhesus macaque (1427)  SLSSWTPVEPATASPASPGGHANPRGNAQRHSQSRASKEGPGQ--------------
                     Dog (1446)  SLSSWTQGDPTAS-AAPLGDHANPRGDTEQHSQCRVSREVLGTVSTEHPHSECL---
                     Pig (1438)  SLSSWTQGDPTS--PSPPGDHANPRGDTERPGKDES---------------------
            Gallus gallus (1427)  SMKLWHCNLLSSCLSSYCFQCVNKSLSELFIYFIFKMVFRILLLPVELKGTVLRTWH
          Xenopus laevis  (1467)  SMTSWNQIEGQLLDLNGSGSQAIVNSSLQLIDQNLNNCDPGPAEQ------------
                Zebrafish (1464)  LASPEDELQKLGLHDSSESSNKEDKEEMLPS--------------------------
 Drosophila melanogaster (1639)  VKDQLAQITDIPKVVELQHRLEAETAEREEAQNKLAVVTGRLDEITRELDNARLEHG
                Consensus (1654)  SLSSW QIE      S  G HA PRGD      Q     E
```

```
                                                                        Section 31
                         (1711) 1711      1720      1730      1740      1750         1767
           Homo sapiens (1499) ---------------------------------------------------------
           Mus musculus (1488) ---------------------------------------------------------
        Rhesus macaque (1470) ---------------------------------------------------------
                    Dog (1499) ---------------------------------------------------------
                    Pig (1472) ---------------------------------------------------------
           Gallus gallus (1484) NSKCRMFCSKGLFETSL----------------------------------------
         Xenopus laevis (1512) ---------------------------------------------------------
              Zebrafish (1495) ---------------------------------------------------------
Drosophila melanogaster (1696) AQILRMEETAREVGNKNAELCELIEFYRNRVEALERLLLASNQELEELNSIQSNQAE
              Consensus (1711)
                                                                        Section 32
                         (1768) 1768        1780      1790      1800      1810         1824
           Homo sapiens (1499) ---------------------------------------------------------
           Mus musculus (1488) ---------------------------------------------------------
        Rhesus macaque (1470) ---------------------------------------------------------
                    Dog (1499) ---------------------------------------------------------
                    Pig (1472) ---------------------------------------------------------
           Gallus gallus (1501) ---------------------------------------------------------
         Xenopus laevis (1512) ---------------------------------------------------------
              Zebrafish (1495) ---------------------------------------------------------
Drosophila melanogaster (1753) GVRDLGDTYSAAEGRQTESDQDKERYQKLALDCKILQAKYRDAKDEIKRCEKKIKDQ
              Consensus (1768)
                                                                        Section 33
                         (1825) 1825 1830      1840      1850      1860      1870       1881
           Homo sapiens (1499) ---------------------------------------------------------
           Mus musculus (1488) ---------------------------------------------------------
        Rhesus macaque (1470) ---------------------------------------------------------
                    Dog (1499) ---------------------------------------------------------
                    Pig (1472) ---------------------------------------------------------
           Gallus gallus (1501) ---------------------------------------------------------
         Xenopus laevis (1512) ---------------------------------------------------------
              Zebrafish (1495) ---------------------------------------------------------
Drosophila melanogaster (1810) RLEMEGKLEKMKNKMKGSRRRRWPQLMLLFIGLIIILIVFFLYYILYINGINVIFKSR
              Consensus (1825)
                                                                        Section 34
                         (1882) 1882 1886
           Homo sapiens (1499) -----
           Mus musculus (1488) -----
        Rhesus macaque (1470) -----
                    Dog (1499) -----
                    Pig (1472) -----
           Gallus gallus (1501) -----
         Xenopus laevis (1512) -----
              Zebrafish (1495) -----
Drosophila melanogaster (1867) SPIVY
              Consensus (1882)
```
