# Supplementary figures and images for: Mutation of S461, in the GOLGA3 phosphorylation site, does not affect mouse spermatogenesis
Source: PeerJ. 2023 Apr 17;11:e15133. doi: 10.7717/peerj.15133 (PMC10117384; doi:10.7717/peerj.15133)

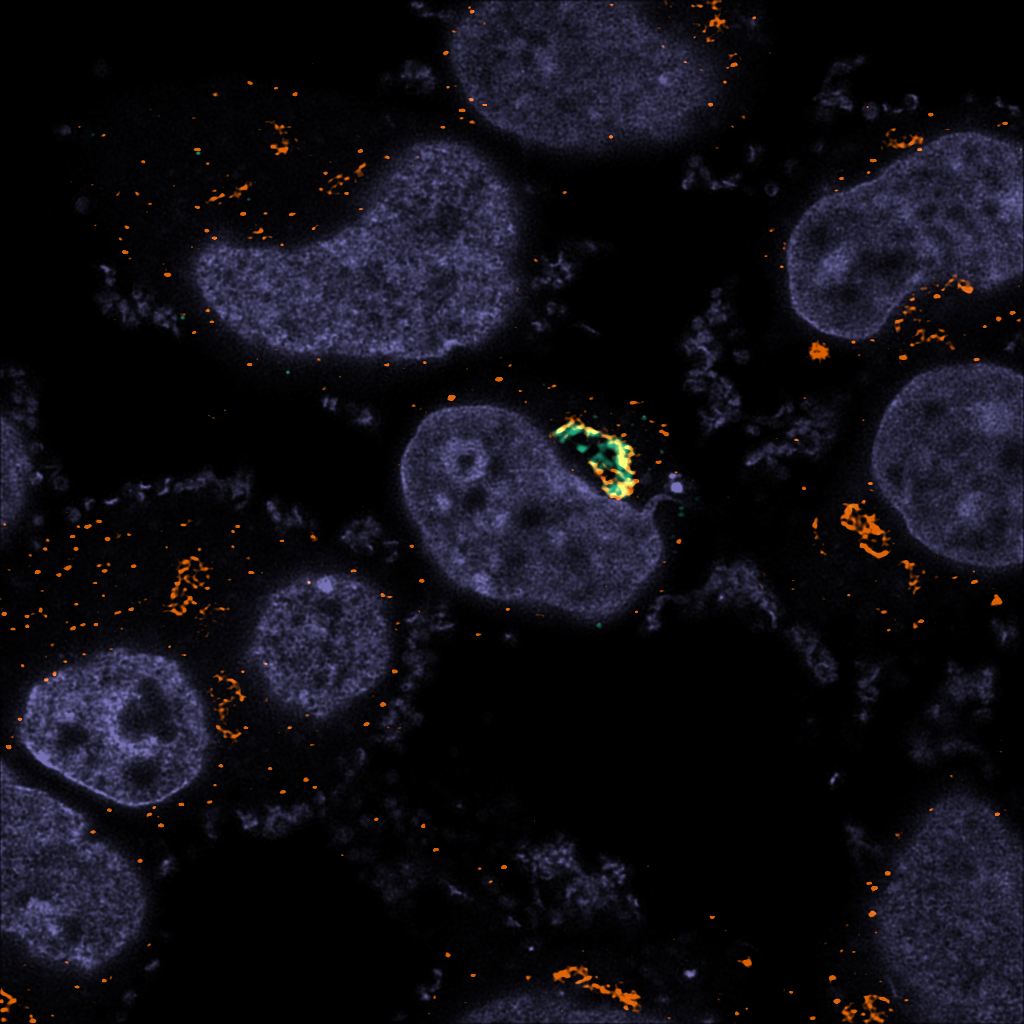

Supplement: Figure S1 — PZFX is a Prism file form and can be opened using GraphPad Prism 5.0 or later. We used GraphPad Prism 6.0. [file peerj-11-15133-s005.zip › Fig1_Raw_data/figure1-Raw data/GOLGA3-GM130/272_Series005_Lng.tif]

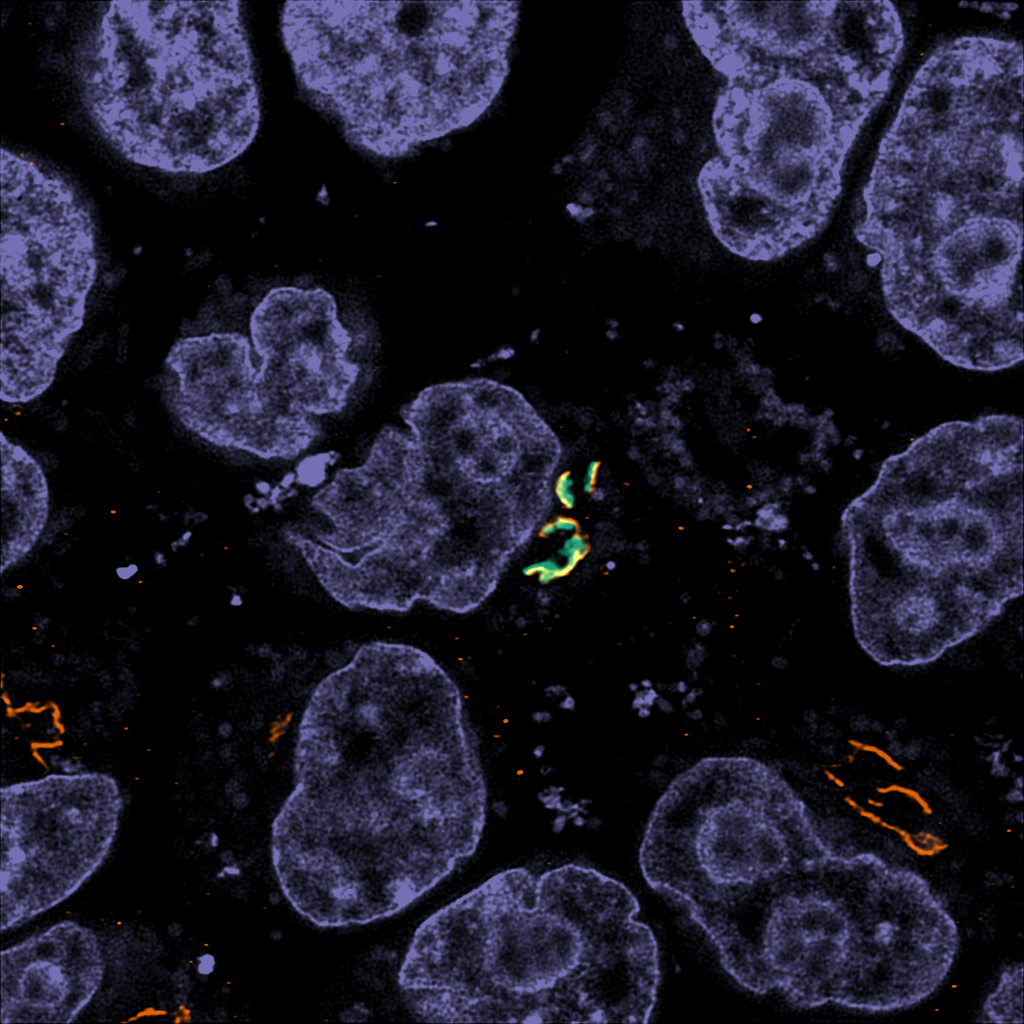

Supplement: Figure S1 — PZFX is a Prism file form and can be opened using GraphPad Prism 5.0 or later. We used GraphPad Prism 6.0. [file peerj-11-15133-s005.zip › Fig1_Raw_data/figure1-Raw data/GOLGA3-GM130/385_Series001_Lng.tif]

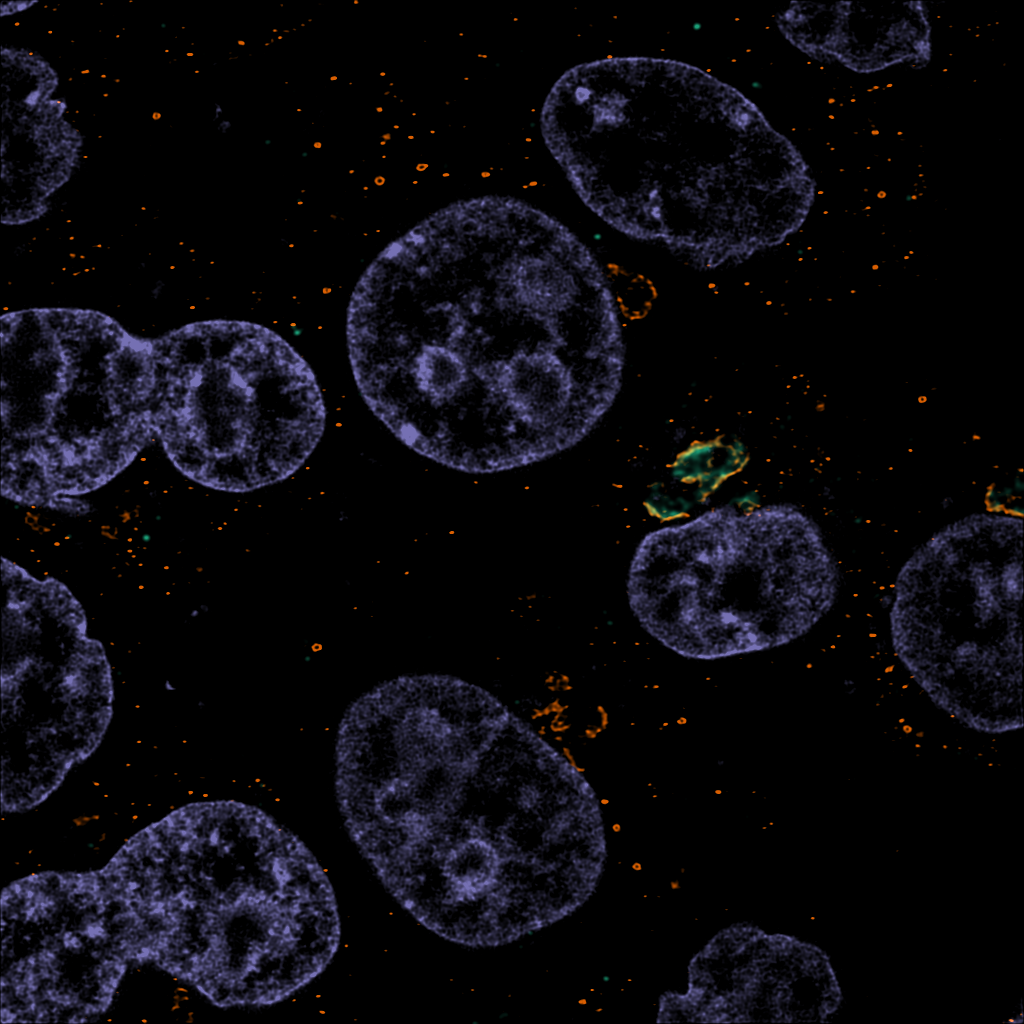

Supplement: Figure S1 — PZFX is a Prism file form and can be opened using GraphPad Prism 5.0 or later. We used GraphPad Prism 6.0. [file peerj-11-15133-s005.zip › Fig1_Raw_data/figure1-Raw data/GOLGA3-GM130/389_Series010_Lng.tif]

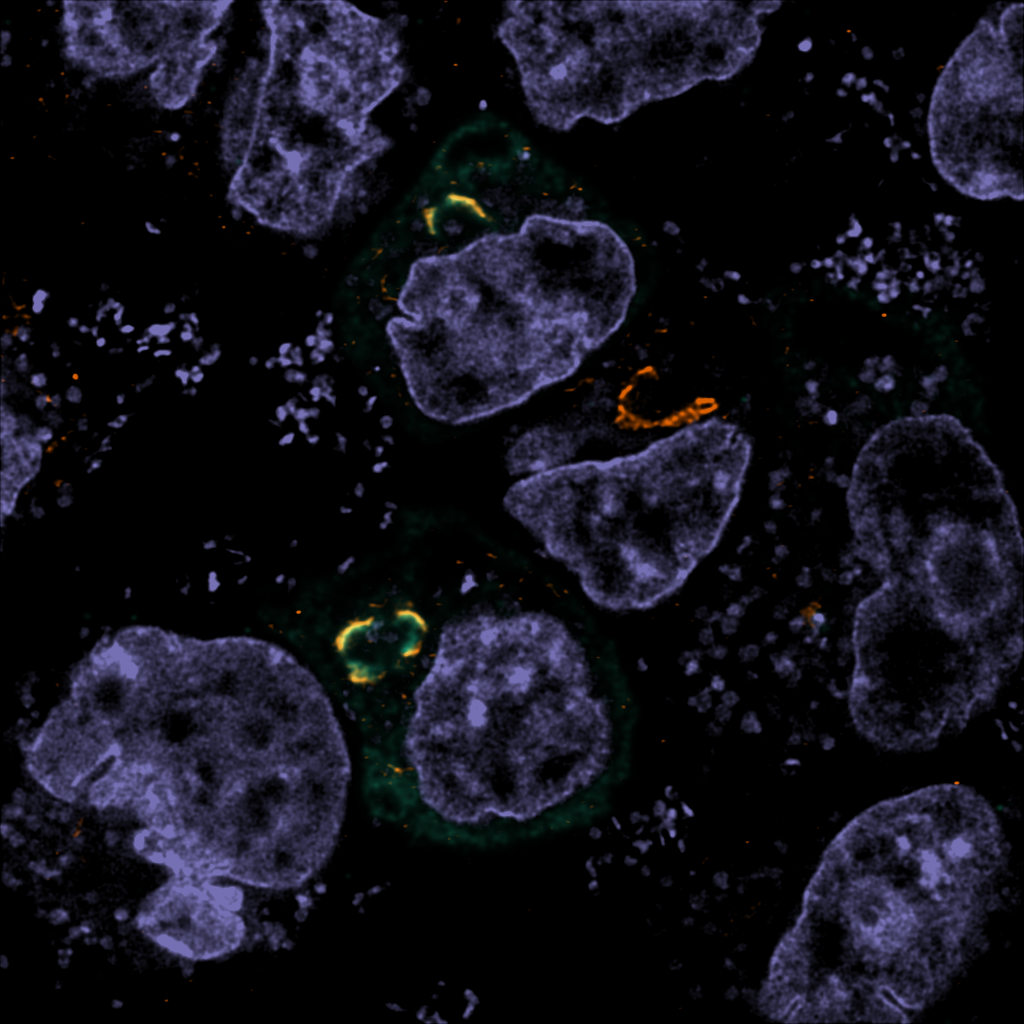

Supplement: Figure S1 — PZFX is a Prism file form and can be opened using GraphPad Prism 5.0 or later. We used GraphPad Prism 6.0. [file peerj-11-15133-s005.zip › Fig1_Raw_data/figure1-Raw data/GOLGA3-GM130/465_Series002_Lng.tif]

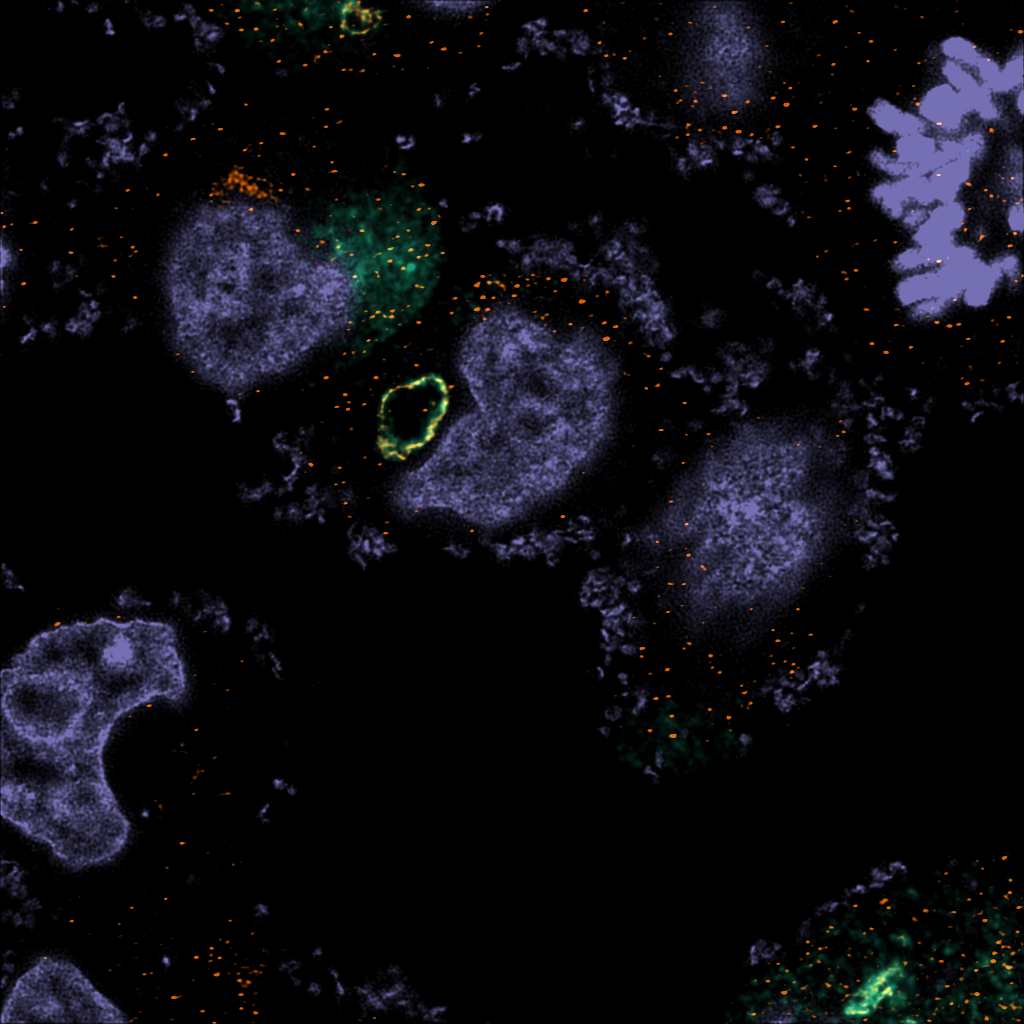

Supplement: Figure S1 — PZFX is a Prism file form and can be opened using GraphPad Prism 5.0 or later. We used GraphPad Prism 6.0. [file peerj-11-15133-s005.zip › Fig1_Raw_data/figure1-Raw data/GOLGA3-GM130/983_Series012_Lng.tif]

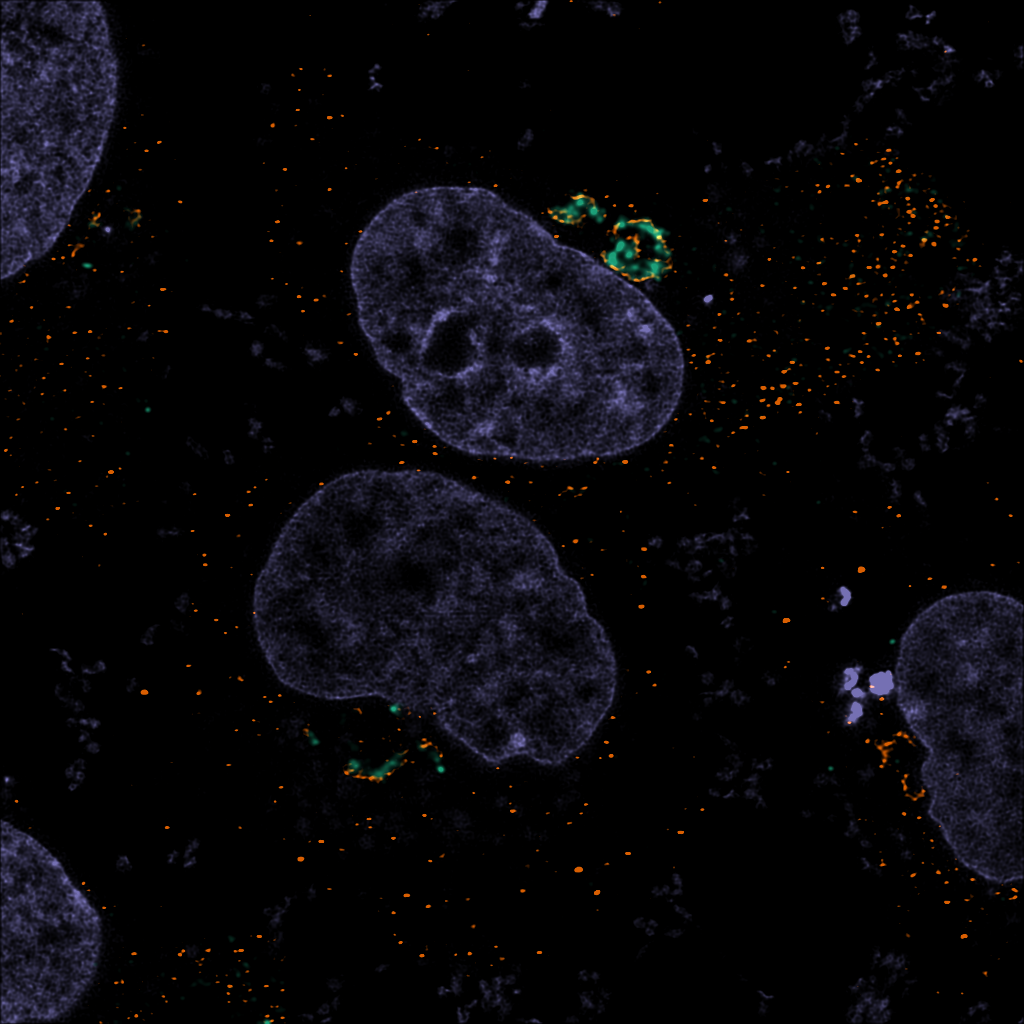

Supplement: Figure S1 — PZFX is a Prism file form and can be opened using GraphPad Prism 5.0 or later. We used GraphPad Prism 6.0. [file peerj-11-15133-s005.zip › Fig1_Raw_data/figure1-Raw data/GOLGA3-GM130/cds2_Series002_Lng.tif]

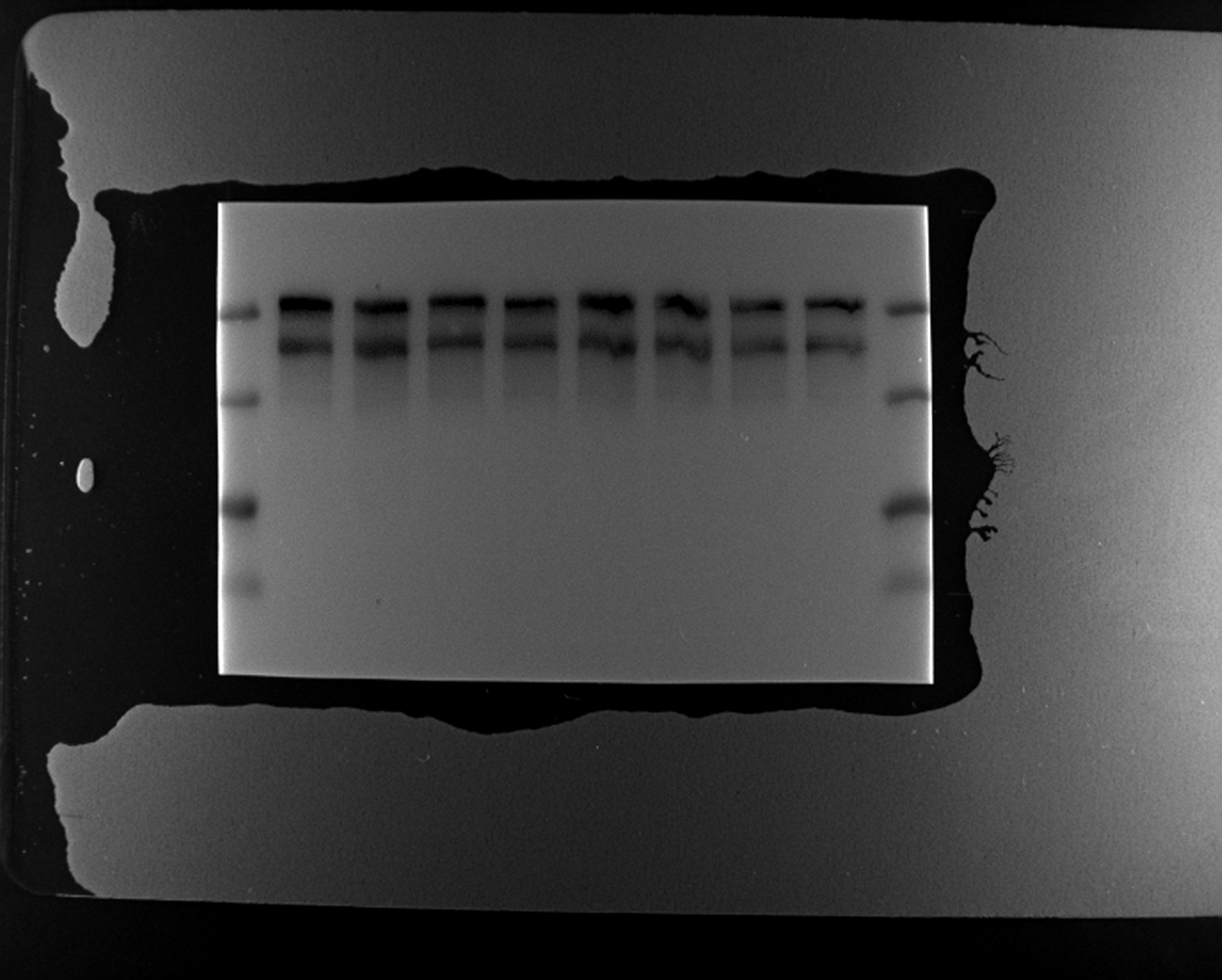

Supplement: Supplemental Information 10 [file peerj-11-15133-s010.tif]

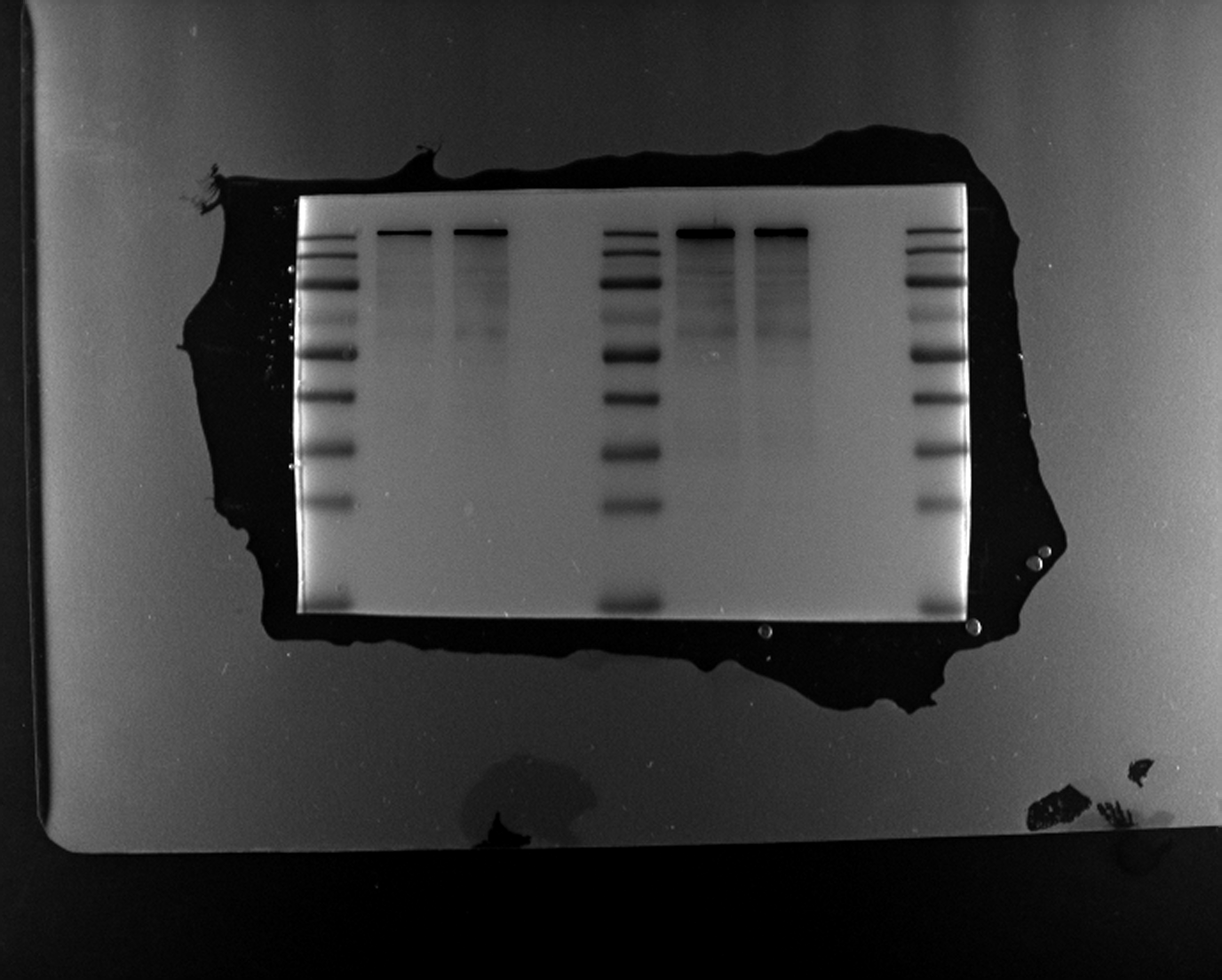

Supplement: Supplemental Information 12 [file peerj-11-15133-s012.tif]
